# A comprehensive spatio-cellular map of the human hypothalamus

**DOI:** 10.1038/s41586-024-08504-8

**Published:** 2025-02-05

**Authors:** John A. Tadross, Lukas Steuernagel, Georgina K. C. Dowsett, Katherine A. Kentistou, Sofia Lundh, Marta Porniece, Paul Klemm, Kara Rainbow, Henning Hvid, Katarzyna Kania, Joseph Polex-Wolf, Lotte Bjerre Knudsen, Charles Pyke, John R. B. Perry, Brian Y. H. Lam, Jens C. Brüning, Giles S. H. Yeo

**Affiliations:** 1 Medical Research Council Metabolic Diseases Unit, Institute of Metabolic Science-Metabolic Research Laboratories, University of Cambridge, Cambridge, UK; 2 Cambridge Genomics Laboratory, Cambridge University Hospitals NHS Foundation Trust, Cambridge, UK; 3 Department of Histopathology, Cambridge University Hospitals NHS Foundation Trust, Cambridge, UK; 4 Department of Neuronal Control of Metabolism, Max Planck Institute for Metabolism Research, Cologne, Germany; 5 Medical Research Council Epidemiology Unit, Institute of Metabolic Science, University of Cambridge, Cambridge, UK; 6 Research & Early Development, Novo Nordisk A/S, Måløv, Denmark; 7 Genomics Core, Cancer Research UK Cambridge Institute, Cambridge, UK; 8 Cologne Excellence Cluster on Cellular Stress Responses in Aging-Associated Diseases (CECAD) and Center for Molecular Medicine Cologne (CMMC), University of Cologne, Cologne, Germany; 9 Center for Endocrinology, Diabetes and Preventive Medicine (CEDP), University Hospital Cologne, Cologne, Germany; 10 National Center for Diabetes Research (DZD), Neuherberg, Germany

**Keywords:** Neural circuits, Obesity

## Abstract

The hypothalamus is a brain region that plays a key role in coordinating fundamental biological functions^1^. However, our understanding of the underlying cellular components and neurocircuitries have, until recently, emerged primarily from rodent studies^2,3^. Here we combine single-nucleus sequencing of 433,369 human hypothalamic cells with spatial transcriptomics, generating a comprehensive spatio-cellular transcriptional map of the hypothalamus, the ‘HYPOMAP’. Although conservation of neuronal cell types between humans and mice, as based on transcriptomic identity, is generally high, there are notable exceptions. Specifically, there are significant disparities in the identity of pro-opiomelanocortin neurons and in the expression levels of G-protein-coupled receptors between the two species that carry direct implications for currently approved obesity treatments. Out of the 452 hypothalamic cell types, we find that 291 neuronal clusters are significantly enriched for expression of body mass index (BMI) genome-wide association study genes. This enrichment is driven by 426 ‘effector’ genes. Rare deleterious variants in six of these (*MC4R*, *PCSK1*, *POMC*, *CALCR*, *BSN* and *CORO1A*) associate with BMI at population level, and *CORO1A* has not been linked previously to BMI. Thus, HYPOMAP provides a detailed atlas of the human hypothalamus in a spatial context and serves as an important resource to identify new druggable targets for treating a wide range of conditions, including reproductive, circadian and metabolic disorders.

## Abstract (web summary)

HYPOMAP integrates single-nucleus RNA sequencing and spatial transcriptomic data to create a comprehensive spatio-cellular map of the human hypothalamus.

## Main

The hypothalamus plays a key role in coordinating fundamental biological functions, including maintaining body temperature, sleep, thirst and energy homeostasis, as well as regulating sexual and parental behaviour, response to stress and circadian rhythms^1^. Yet, despite its importance, our understanding of its architecture has so far emerged primarily from rodent studies.

Human genetic studies have uncovered many principal components of the hypothalamic appetitive^2^ and reproductive^4^ control pathways. The fat sensing leptin–melanocortin pathway, which comprises pro-opiomelanocortin (POMC) and agouti-related peptide (AgRP) neurons in the hypothalamic arcuate nucleus (ARC), acting through intra- and extra-hypothalamic projections to control food intake and energy expenditure, represents an essential regulatory pathway. We know it plays a key role in the control of appetite because genetic disruption of the pathway results in severe obesity, not only in humans and mice, but also in many other vertebrate species^2,3^. Recently, we found that the leptin–melanocortin pathway also plays important roles in linear growth and pubertal onset, through the melanocortin 3 receptor (MC3R)^5^. However, our understanding of the melanocortin neurocircuitry is derived largely from functional studies in mice^2,3^.

Despite the paucity of human hypothalamic studies, currently licensed therapies for the treatment of obesity and diabetes, including semaglutide^6^ and tirzepatide^7^, target the hypothalamus^8^. Semaglutide is a long-acting glucagon-like peptide-1 receptor (GLP1R) agonist and tirzepatide is a GLP1R/glucose-dependent insulinotropic polypeptide receptor (GIPR) co-agonist; both are thought to mediate their effects on energy intake, at least in part, through POMC neurons^8^. Nevertheless, studies supporting the more detailed molecular modes of action are derived from studies in mice^8–10^. Additionally, setmelanotide—an MC4R agonist—has recently been approved for treating rare genetic causes of obesity^11^.

Given the therapeutic focus on the hypothalamus, enhancing our understanding of its human-specific architecture is crucial. Here we have integrated single-nucleus RNA sequencing (snRNA-seq) and spatial transcriptomic data to create a comprehensive spatio-cellular map of the human hypothalamus.

## HYPOMAP captures more than 430,000 cells

**Fig. 1:**
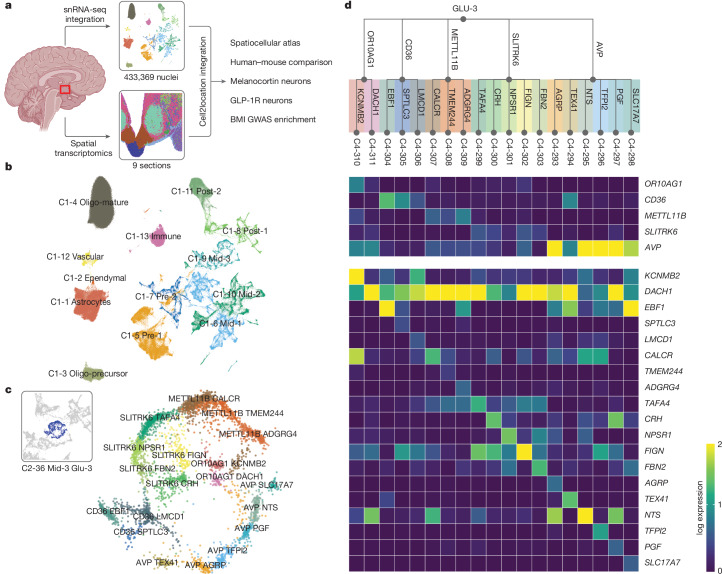
Integrated snRNA-seq reference atlas of the human hypothalamus. **a**, Schema of datasets and analyses results. **b**, UMAP plot of the integrated atlas coloured by principal cell classes. Colouring corresponds to background colours in Fig. 2. **c**, Details of cluster C2-36 Mid-3 GLU-3. This glutamatergic *SIM1*-expressing cluster includes AVP neurons of the PVN as well as subclusters from mammillary bodies. Inset, position on the global UMAP that is expanded and coloured by the C4 subcluster. **d**, Dendrogram of C2-36 Mid-3 GLU-3 with a detailed overview of the subcluster structure. Colouring corresponds to the UMAP in **c**. The heatmap shows the average expression (in log-normalized scale) of marker genes used for annotation of C3 and C4 clusters. Illustration in **a** created using BioRender (https://biorender.com); credit: S. O’Rahilly (2024).

**Extended Data Fig. 1:**
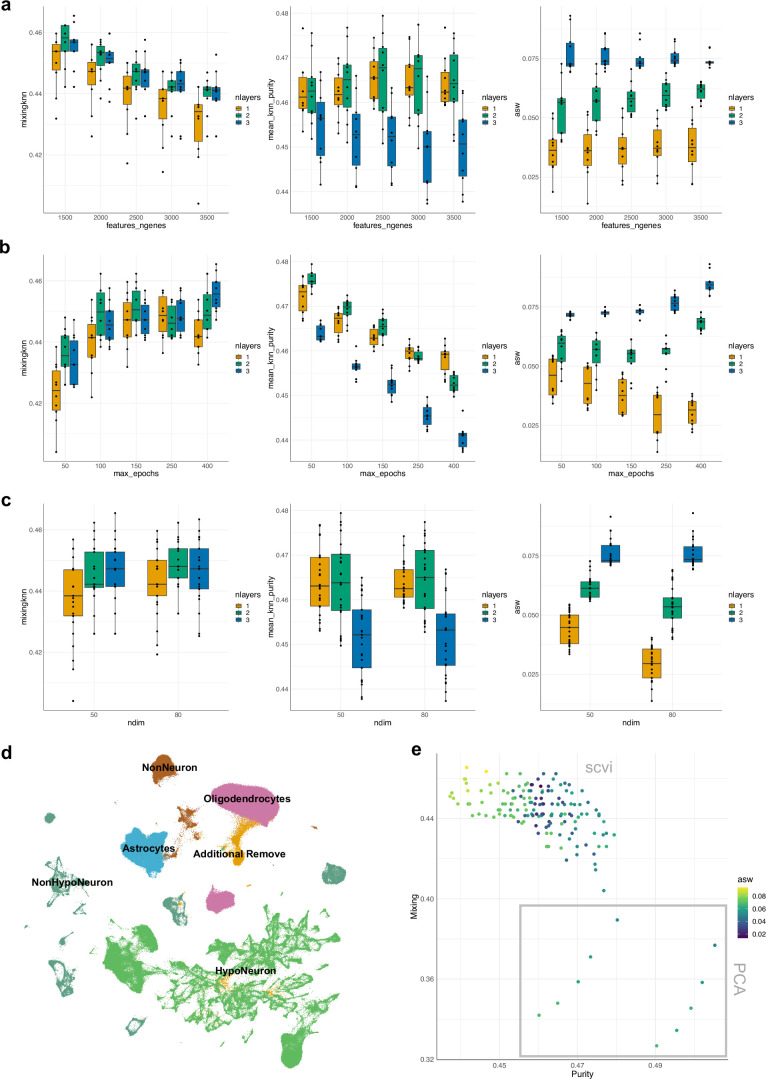
snRNAseq data integration. **a-c**, Evaluation metrics for scvi integration results of the combined dataset (see Methods for a brief explanation of metrics) used to select the final set of hyperparameters. Boxplots show evaluation metrics of different hyperparameters runs coloured by the number of layers in the model and stratified by the HVG set size (**a**), the number of training epochs (**b**) and the number of latent space dimensions (**c**). Each point corresponds to an scvi training run (n = 150). The centre of each boxplot corresponds to the median, the lower and upper hinges correspond to the first and third quartiles and the whiskers extend from each hinge to the largest (or smallest) value smaller (or larger) than 1.5 times the distance between the first and third quartiles. **d**, UMAP plot of the dataset after integration but before removal of additional cell types such as non-hypothalamic neurons. **e**, Scatter plot of purity vs mixing metric coloured by average silhouette width of all runs. PCA without further integration achieved good purity but did not integrate the data as well as scvi.

**Extended Data Fig. 2:**
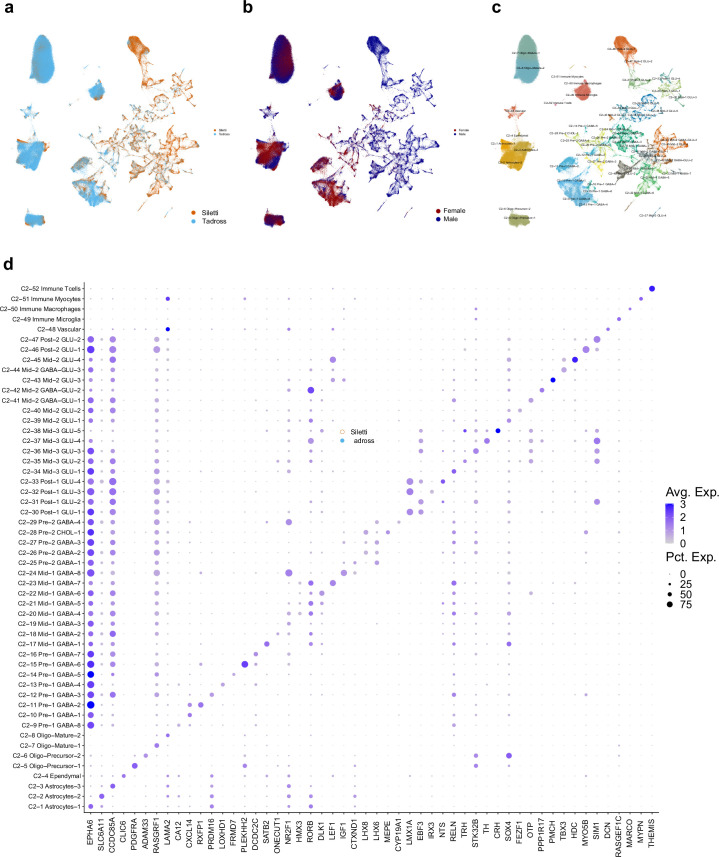
snRNA-seq reference atlas of the human hypothalamus. **a**, UMAP plot of the integrated atlas coloured by the two contributing studies. **b**, UMAP plot of the integrated atlas coloured by donor sex. **c**, UMAP plot of the integrated atlas coloured by clustering level C2. **d**, Dotplot of top marker genes per cluster on level C2 vs all other cells within the same C0 cluster. Dot size corresponds to the percentage of expressing cells in that cluster and colour intensity to the average expression level.

**Extended Data Fig. 3:**
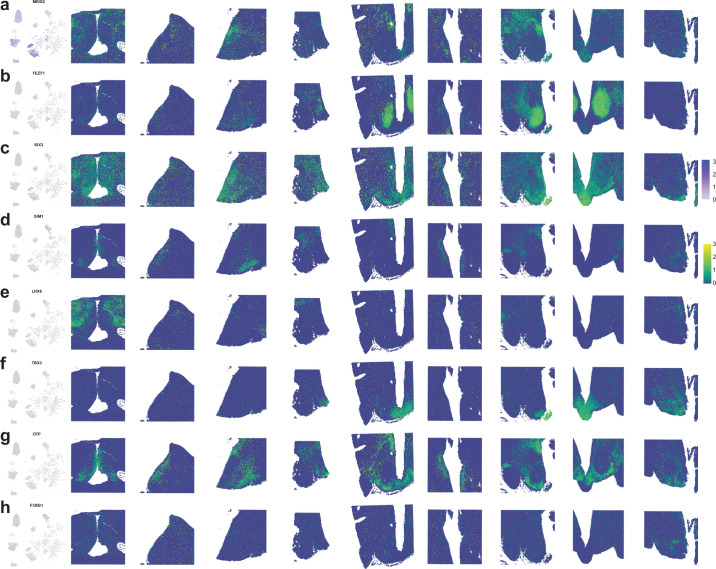
Key transcription factors in the snRNA-seq reference atlas of the human hypothalamus and in the 9 ST sections. **a-h**, UMAP plots showing per cell expression, and spatial expression plots showing per spot expression (log-normalized scale) of the transcription factors *MEIS2*, *LHX6*, *FEZF1*, *TBX3*, *SIX3*, *OTP*, *SIM1* and *FOXB1*.

We collected frozen hemi-hypothalami from eight brain donors of normal body mass index (BMI) (range 18–28 kg m^−2^; details in Supplementary Table 1), and performed snRNA-seq (Fig. 1a; Methods). After quality control steps (Methods), we captured 311,964 nuclei with an average of 4,541 ± 10.6 (mean ± s.e.m.) counts detected per nucleus (mean 2,040 ± 2.4 genes per nucleus). In addition, we extracted the expression matrix for 121,405 nuclei (20,331 ± 55.9 [mean ± s.e.m.] counts detected and 5097 ± 7.1 genes per nucleus) from the hypothalamic regions of three separate donors from a publicly available whole-brain dataset^12^ (Supplementary Tables 1 and 2). We integrated the two datasets using scvi-tools^13,14^, and generated a reference database of the human hypothalamus consisting of a total of 433,369 nuclei that we call ‘HYPOMAP’ (Extended Data Figs. 1 and 2 and Supplementary Tables 3 and 4). A uniform manifold approximation projection (UMAP) plot is shown in Fig. 1b, illustrating the different main cell types identified from the human hypothalamus. The expression of key transcription factors, used as regional markers, demonstrates that our dataset indeed spans the hypothalamus (Extended Data Fig. 3).

## Flexible multi-level cell clustering

**Fig. 2:**
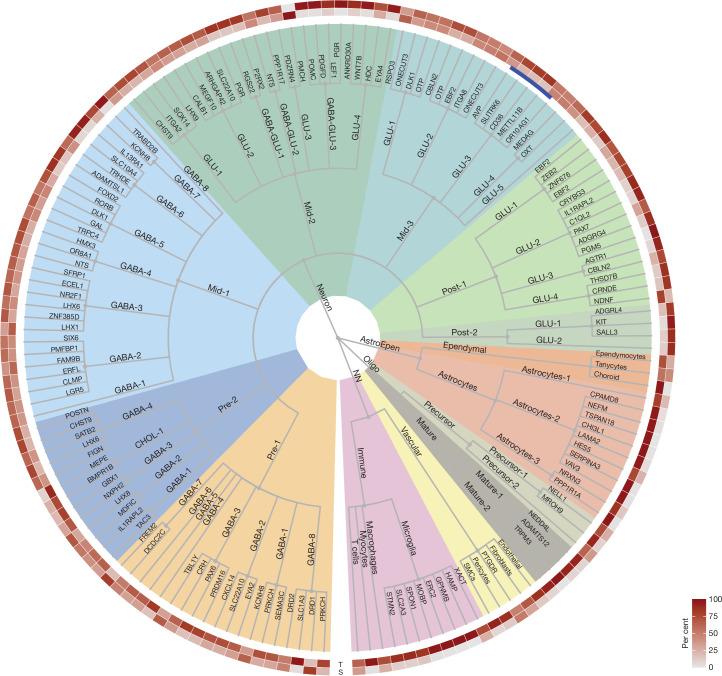
Cell-type taxonomy of the human hypothalamus. Hierarchical consensus tree (circular dendrogram) of human cell types based on unbiased clustering of the integrated snRNA-seq data. Nodes correspond to clusters at each level in the tree. At the highest level (C0) the tree comprises four main subgroups: neurons, Oligo, AstroEpen and other non-neuronal (NN) cells. Subsequent levels C1–C3 further define the cell-type structure in the human hypothalamus. For neurons, a further level C4 with up to 452 total clusters exists (not shown). Edges are labelled with cluster names. The heatmap ring depicts the relative contribution of cells by the two studies to each cluster on level C3. Further information on tree structure, cluster annotation and marker genes can be found in Supplementary Tables 5–8.

HYPOMAP comprises 166,475 neurons; 175,109 oligodendrocytes (Oligo); 63,111 astro-ependymal cells (AstroEpen) and 28,674 cells from other non-neuronal cell types, including microglia and endothelial cells (Fig. 1b). We adopted multi-level hierarchical clustering by coupling the Leiden, consensus clustering and multi-resolution tree (mrtree) algorithms to enable flexibility in cell-type classification and ensure optimal granularity for downstream analyses (Methods). The final clustering tree, shown as a circular dendrogram in Fig. 2, consists of five levels and 452 clusters (levels C0–C3 shown in Fig. 2 to preserve visibility, C0 = 4, C1 = 13, C2 = 52, C3 = 156, C4 = 452 clusters). Neuronal clusters are annotated based on broad anatomical location (C1), neurotransmitter class (C2) and informative marker genes (C3 and C4). Non-neuronal clusters are labelled by their common names and marker genes. Individual cluster annotations are indicated on the edges of the tree in Fig. 2. Most neuronal cell types located in the middle of the hypothalamus (along the anterior–posterior axis), are organized in the clusters Mid-1 to Mid-3 and include neurons of the ARC, ventromedial hypothalamus (VMH), suprachiasmatic nucleus (SCN) and paraventricular nucleus (PVN) (Figs. 1b and 2 and Supplementary Tables 5–8). Supplementary Table 9 includes a mapping of our C2–C4 clusters to a recently published description of developmental and adult human cell types^15^.

To illustrate the functionality of our atlas, focus on a subset of glutamatergic *SIM1-*expressing neurons at level C2 as an exemplar, highlighted in blue in Figs. 1c and 2. These neurons subcluster into 5 different clusters at the next level C3, and 19 on the most granular level C4. The C4 clusters include a likely PVN magnocellular arginine-vasopressin (AVP) TFPI2 cluster (C4-296), with co-expression of *TH*, *OXT* and *SCGN*—marker genes that are mutually exclusive in mice^16^ (Fig. 1c,d and Supplementary Tables 7–8).

## Spatio-cellular mapping

**Fig. 3:**
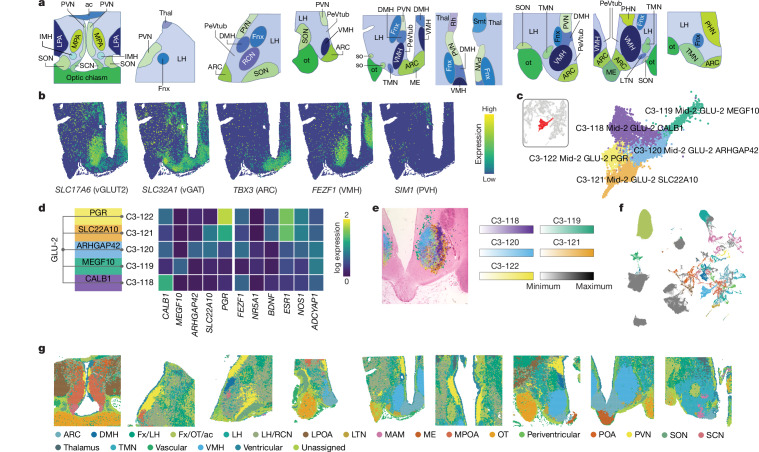
Spatial transcriptomics of the human hypothalamus and mapping of cell clusters identified by snRNA-seq. **a**, Reference atlas diagrams of the nine human hypothalamic sections used for spatial transcriptomics (seven donors) ordered from most anterior (left) to most posterior (right). **b**, log-normalized spatial expression plots of glutamatergic and GABAergic markers *SLC17A6* and *SLC32A1* and transcription factors *TBX3*, *FEZF1* and *SIM1*, used to mark the ARC, VMH and PVN, respectively, in a mid-hypothalamic section. **c**–**e**, Details of five C3 branches of C2-35 Mid-2 GLU-2. **c**, UMAP of C2-35 branch clusters highlighted in red, and the subset of C2-35 coloured and labelled by its five C3 branches. The colouring of the clusters corresponds to **d**. **d**, Dendrogram that summarizes subcluster structure and displays a heatmap of the mean log-normalized expression of each cluster’s marker gene. **e**, Cell2location mapping of the five C3 clusters to subregions of the VMH in a mid-posterior hypothalamic section. **f**, snRNA-seq UMAP plot coloured by the spatial region to which each C3 neuronal and C2 non-neuronal cluster was assigned (Methods). Colours are consistent with colours and labelling in **g**. **g**, Spatial plots displaying Leiden clustering of spots from the co-occurrence of snRNA-seq cluster abundance scores. Spots in the same cluster show similar patterns of snRNA-seq cell abundance. Clusters were named and grouped based on concordance to established hypothalamic neuroanatomical structures. A list of abbreviations can be found in Supplementary Table 20.

Using Visium technology (10x Genomics), we performed spatial transcriptomic profiling of nine hypothalamic sections from seven donors, covering the preoptic/anterior, middle and the posterior hypothalamus (regional annotation shown in Fig. 3a, atlas location found in Supplementary Table 10). An example can be seen in a mid-hypothalamic section, where we can discriminate spatially restricted expression of *SLC17A6* and *SLC32A1*, corresponding to glutamatergic VMH neurons and GABAergic ARC neurons respectively (Fig. 3b). The region-specific expression of transcription factors *TBX3*, *FEZF1* and *SIM1*, which mark the ARC, VMH and PVN, respectively, are also clearly illustrated (Fig. 3b).

Because Visium does not achieve single-cell resolution (each spot typically covering one to ten cells^17^), we integrated the spatial transcriptomic and snRNA-seq data using cell2location—a Bayesian model-based deconvolution package^18^—to spatially map snRNA-seq cell clusters to the hypothalamic sections shown in Fig. 3a. Figure 3c–e showcases the spatial mapping of VMH cell clusters identified in the snRNA-seq data. We identify five VMH neuronal clusters at C3 (Fig. 3c,d) and 23 at C4, respectively, expressing known VMH-elated genes such as *ESR1*, *BDNF, NR5A1* and *ADCYPAP1* (ref. ^19^). All five C3 clusters show spatially distinct mapping in the VMH (Fig. 3e).

To identify tissue regions to which sets of snRNA-seq clusters consistently map, we performed Leiden clustering on the cell abundance values of each cluster in each Visium spot (C3 for neuronal clusters, C2 for non-neuronal clusters). This yielded 27 clusters that we further grouped based on the hypothalamic region where most spots are located, resulting in 23 regional clusters. Of note, we identify clusters for the ARC, VMH, SCN, PVN, SON, lateral tuberal nucleus (LTN), median eminence (ME) and tuberomammillary nucleus (TMN), as well as clusters in predominantly non-neuronal regions, for example, the optic tract. Using this, we assigned regional annotations to each snRNA-seq neuronal C3 cluster and non-neuronal C2 cluster based on abundance scoring in each region (Fig. 3f,g and Supplementary Tables 11–13; Methods).

## Non-neuronal spatial heterogeneity

**Extended Data Fig. 4:**
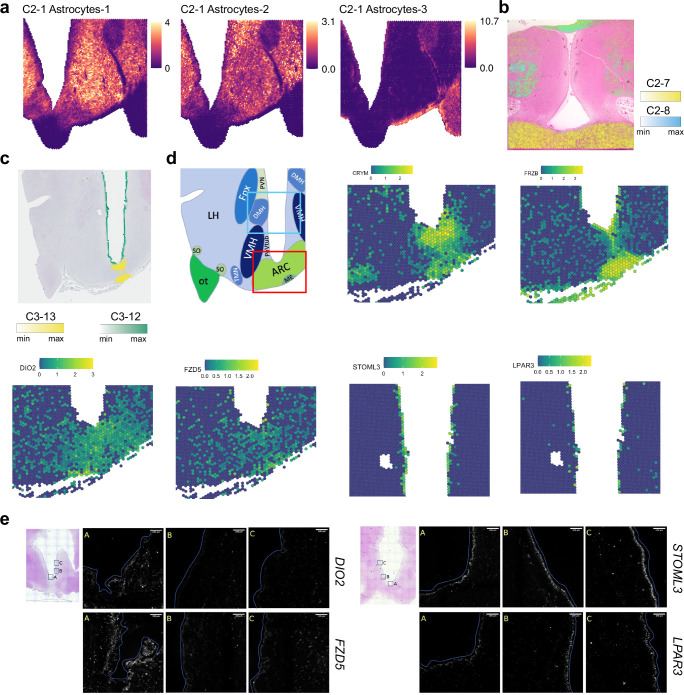
Non-neuronal cell types in the human hypothalamus. **a**, Spatial mapping of astrocyte populations at the C2 cluster level: cell2location mapping of each of the 3 astrocyte C2 clusters, demonstrating spatially distinct astrocytic populations. **b**, Spatial mapping of oligodendrocyte populations at the C2 level in the anterior hypothalamus. Cell2location maps the two C2 oligodendrocyte populations to distinct locations in the hypothalamus: C2-8 oligodendrocytes map to the anterior commissure, and C2-7 oligodendrocytes map to the optic chiasm, with both cell types mapping to the lateral preoptic area. **c**, Mapping of ependymal cell types in the hypothalamus. Cell2location mapping of tanycytes (yellow) and ependymal cell (green) types in the hypothalamus. Tanycytes show distinct mapping to the median eminence and the base of the third ventricle, whereas ependymal cells map to the lining of the third ventricle. **d**, Log-normalized expression of *CRYM* and *FRZB*, both previously identified to be markers of tanycyte subtypes, in the third section below the third ventricle. (Below) Log-normalized expression of *DIO2*, *FZD5*, *STOML3* and *LPAR3*, marker genes for tanycytes and/or ependymal cells with **(e)** complementary smFISH. For each probe the assay was successfully repeated between 1 and 3 times on 3 biological replicates.

Non-neuronal cells have been historically understudied and, although less heterogeneous than their neuronal counterparts, still show considerable diversity as revealed by single-cell approaches^20^. At C2, hypothalamic astrocytes are divided into three main populations showing spatially distinct distributions (Extended Data Figs. 2d and 4a). Similarly, we find spatially restricted populations of oligodendrocytes, ependymocytes and tanycytes (Extended Data Fig. 4b,c).

Campbell and colleagues^21^ previously demonstrated discrete subpopulations of tanycytes in the mouse hypothalamus. Although our snRNA-seq data did not capture sufficient tanycytes (102 cells) to differentiate between subtypes in humans, we did observe different patterns of expression of *CRYM* and *FRZB* in the median eminence (Extended Data Fig. 4d). We also looked further into expression of tanycyte and ependymal marker genes in the spatial transcriptomics. Here we found concentrated expression of *DIO2* and *FZD5* below the third ventricle and in the median eminence region; however, *STOML3* and *LPAR3* show distinct expression in spots lining the entirety of the walls of the third ventricle, indicating that these represent ependymal cell markers (Extended Data Fig. 4d). Further, we confirmed these findings using single-molecule fluorescence in situ hybridization (smFISH) (Extended Data Fig. 4e).

## Human–mouse neuronal conservation

**Fig. 4:**
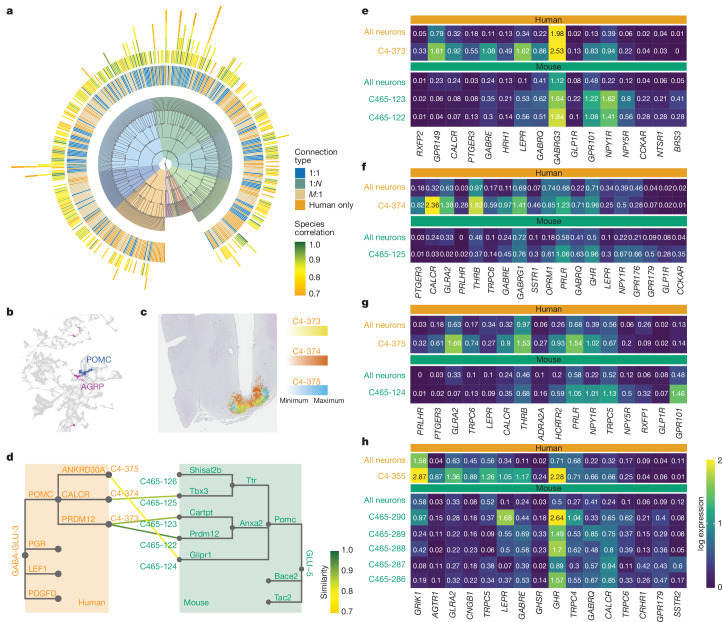
POMC and AgRP neurons across species. **a**, Global comparison of neuronal conservation between human and mouse. The dendrogram corresponds to Fig. 2 but without labels and extended by neuronal clusters on level C4. The inner heatmap depicts the type of cluster cross-species relationship. The outer heatmap is coloured by the correlation of the matched mouse clusters of each human cluster on level C4. Multiple heatmap tiles indicate multiple corresponding mouse clusters (highest first). **b**, UMAP plot highlighting three clusters with the highest percentage of *POMC*^+^ (blue) or *AGRP*^+^ (pink) nuclei. **c**, Spatial mapping of POMC clusters. Cell abundance scores for C4-373 (blue), C4-374 (orange) and C4-375 (yellow) in a section of the human hypothalamus. All three clusters map to the ARC. **d**, Left, dendrogram of human POMC neurons; right, dendrogram of corresponding clusters from mouse HypoMap. Edges are labelled with marker genes used to annotate clusters. The lines connect corresponding cluster between species, highlighting 1:1 and 1:*N* relationships; line colour depicts Pearson correlation. **e**–**h**, Heatmaps showing receptor gene expression average (log-normalized scale) in human POMC and AgRP clusters and their corresponding mouse clusters. Up to ten receptor genes (based on marker gene detection) were included per species. The first row of both facets depicts the average expression in all neurons of the species for comparison: C4-373, Mid-2 GABA-GLU-3 POMC PRDM12 (**e**); C4-374, Mid-2 GABA-GLU-3 POMC CALCR (**f**); C4-375, Mid-2 GABA-GLU-3 POMC ANKRD30A (**g**); C4-355, Mid-2 GABA-GLU-1 RGS22 AGRP (**h**).

**Extended Data Fig. 5:**
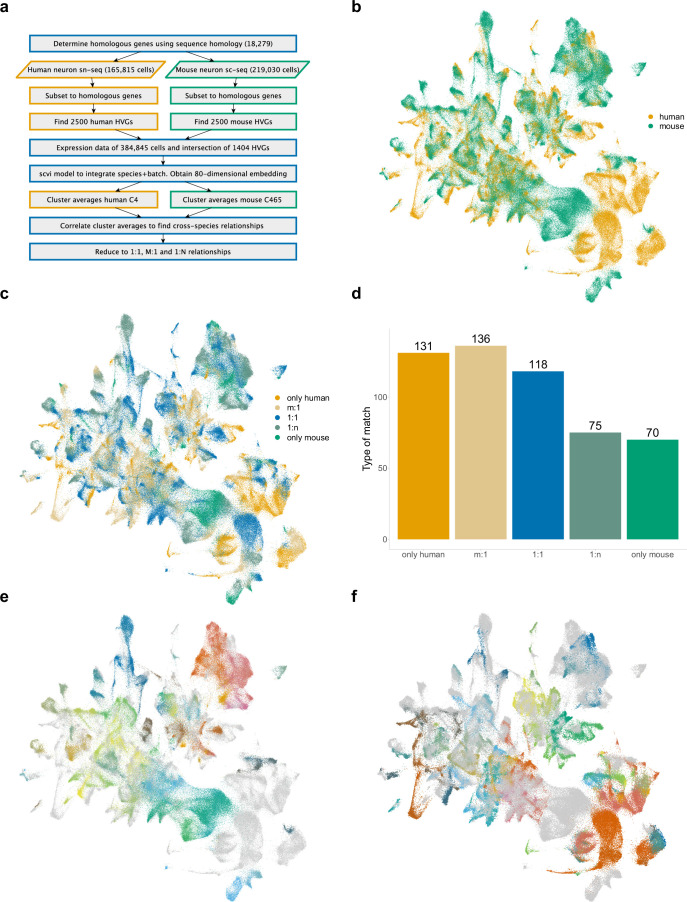
Cross-species integration. **a**, Schema of cross-species integration and cluster comparison approach employed to determine corresponding human and mouse clusters. **b-c**, UMAP plots based on the cross-species integrated scvi embedding using only neuronal cells coloured by species (**b**) and type of cluster relationship (**c**). **d**, Barplot showing the total number for each type of cluster relationship across species. Colours correspond to **c** and Fig. 4a. **e-f**, Same UMAP plots as in **b**, but coloured either by human clusters (C4) (**e**) or by mouse clusters (C465) (**f**). Grey cells indicate cells from the respective other species.

We previously generated a unified mouse hypothalamic single-cell atlas^14^, and here sought to compare the neuronal clusters across the two species, taking a conservative approach by restricting the analysis to homologous genes. We matched the human and mouse clusters on the highest available clustering resolution through the correlations of the C4 (human) and C465 (mouse) cluster averages in scvi embedding obtained by integrating all human and mouse neurons (165,815 and 219,030 cells, respectively) (Fig. 4a and Extended Data Fig. 5). At this level there are 413 human and 320 mouse neuronal clusters. Figure 4a illustrates the conservation between species, where the correlation with matched mouse clusters for each human cluster is shown in the outer heatmap around the tree. The colour reflects correlation strength and indicates the reliability of cell-type matching, and the inner heatmap depicts the type of cross-species relationship (1:*N*, *M*:1 or 1:1, where *N* or *M* indicate a match to more than one cluster, as detailed in Supplementary Table 14). Of the 413 human clusters, 131 (32%) could not be matched to a mouse cluster and 70 of 320 (22%) mouse clusters had no corresponding human cluster, partly due to differences in anatomical scope of each dataset. Of Mid-2 subcluster neurons, well sampled in both datasets and spanning the ARC, VMH the dorsomedial hypothalamus (DMH), 30 of 91 (33%) are human specific. These include various subtypes of *PPP1R17*-expressing neuron of the DMH where heterogeneity seems greater in humans than in mice (Supplementary Table 14).

## Human hypothalamic melanocortin system

**Extended Data Fig. 6:**
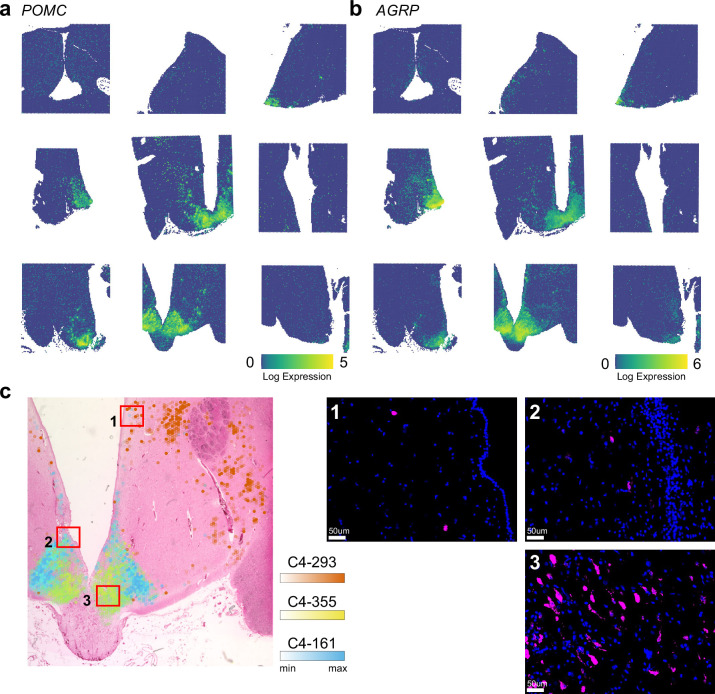
Mapping the leptin-melanocortin pathway: POMC and AgRP. **a-b**, Spatial expression of *POMC* (**a**) and *AGRP* (**b**) (log-normalized) in each of the 9 human hypothalamus sections. **c**,**Left**: Spatial mapping of AgRP clusters. Cell abundance scores from cell2Location for C4-355 (yellow), C4-161 (blue) and C4-293 (orange) and **Right**: smFISH of *AGRP* expression in the mid-posterior hypothalamus in a near adjacent section to the ST (n = 2).

We then examined the hypothalamic leptin–melanocortin pathway, initially focusing on neurons expressing *POMC*—the gene encoding the melanocortin peptides. At C4, we find three closely related clusters with high expression of *POMC* (Fig. 4b and Extended Data Fig. 6a). C4-373 POMC/PRDM12 has the highest level of *POMC* expression, and *LEPR* is one of the key markers for this cluster (68% *POMC* and *LEPR* co-expression). The spatially mapped POMC clusters show distinct distributions, with the canonical POMC/PRDM12 neurons located adjacent to the median eminence and the POMC/CALCR neurons closest to the third ventricle (Fig. 4c). The POMC/PRDM12 subcluster C4-373 corresponds to the two mouse Anxa2.Pomc subclusters, whereas the POMC/CALCR subcluster C4-374 matches Ttr.Pomc (Fig. 4d). The third human subcluster, C4-375, matched the Glipr1.Pomc cluster, albeit with low correlation, indicating divergence in the transcriptomic identity of this POMC neuronal subtype between humans and mice (Fig. 4d).

Although correlations are useful in identifying conserved clusters between species, they cannot be used to explore conservation of gene expression. To address this, we focus predominantly on G-protein-coupled receptors (GPCRs) given their therapeutic relevance. For each POMC cluster, we selected up to ten species-enriched receptors based on marker gene expression (Fig. 4e–g). Notable receptors with conserved cross-species expression in POMC/PRDM12 neurons include leptin and NPY receptors. Receptors for cholecystokinin (*CCKAR*) and bombesin (*BRS3*) are expressed in mice but not in humans (Fig. 4e). Of note, BRS3 agonists are anorexigenic in mice and have been trialled in humans as a treatment for obesity^22^. Although *GLP1R* is expressed in the human POMC/PRDM12 cluster, we and others have shown previously that *Glp1r*- and *Lepr*-expressing POMC neurons are two distinct populations in mice^14,23^ (Fig. 4e,f). The human POMC cluster C4-374, corresponding to the mouse *Glp1r*-expressing POMC neurons, instead expresses the calcitonin receptor (*CALCR*) (Fig. 4f), highlighting the interspecies heterogeneity of POMC populations, which has direct implications for currently licensed obesity therapies.

We identified one cluster with high expression of the endogenous melanocortin antagonist *AGRP*, C4-355, which co-expresses *NPY* and GABAergic markers, and therefore probably represents canonical AgRP/NPY neurons. *AGRP* expression is detected in ARC cluster C4-161, which co-expresses *GHRH*, *GAL* and *GHSR*, and in AVP neurons near the PVN (C4-293) (Fig. 1d and Extended Data Fig. 6b,c). We validated the presence of *AGRP* expression near the PVN using spatial transcriptomics and smFISH (Extended Data Fig. 6b,c). Cluster C4-355 matches all five subclusters of the mouse cluster ‘C66-46, Agrp.GABA-4’ in the mouse HypoMap. Given the much higher number of AgRP neurons sampled in the mouse data (5,244 cells versus 373 cells in C4-355), the absence of further subclustering in the canonical AgRP neuronal cluster is not necessarily indicative of higher biological complexity in mouse. Notable GPCRs with conserved expression in AgRP neurons include the ghrelin receptor (*GHSR*) and the growth hormone receptor (*GHR*). We also observe a high expression level of the angiotensin II receptor type 1 gene (*AGTR1*) in humans, which is not found in mouse, indicating species-specific roles in metabolic regulation and potential implications for disease (Fig. 4h).

**Extended Data Fig. 7:**
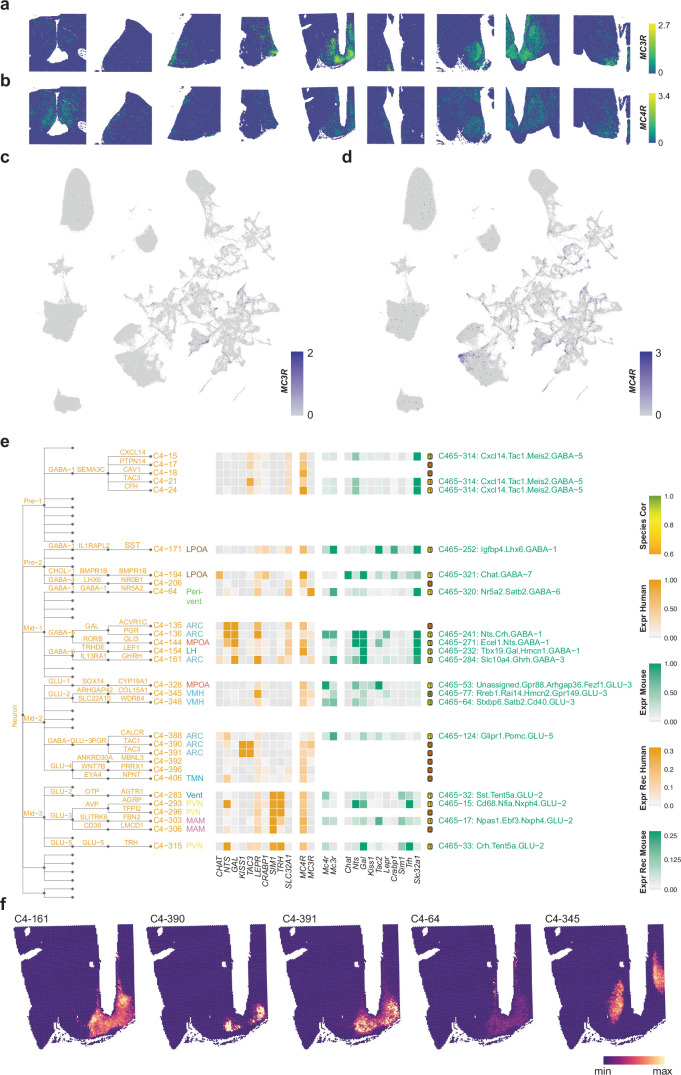
Mapping the leptin-melanocortin pathway: MC3R and MC4R. **a-b**, Log-normalized spatial expression of *MC3R* (**a**) and *MC4R* (**b**) in the human hypothalamus ST sections. **c-d**, UMAP expression plots (log-normalized) showing *MC3R* (**c**) and *MC4R* (**d**) in the snRNA-seq dataset. **e**, Clusters expressing *MC4R* or *MC3R* in either human or mouse. **Left**: The dendrogram depicts the top 5% of all human C4 clusters expressing *MC4R* or *MC3R* and their ancestors on level C1-3. Edges are labelled with cluster names. Regional annotations (if applicable) are annotated next to each C4 cluster. **Right**: The highest correlated mouse cluster (if available) is shown. The small number indicates the total number of matched mouse clusters to the human C4 cluster and is coloured by correlation of the highest cluster. **Middle**: The outer heatmaps depict the average expression of selected marker genes in human (orange) or mouse (green) clusters. The inner heatmaps depict the average expression of *MC4R* and *MC3R* in human (orange) or mouse (green) clusters. **f**, Cell abundance scores from cell2location of 5 *MC3R*-expressing clusters (C4-161, C4-390, C4-391, 4-161, C4-64) in a medial section of the human hypothalamus.

In contrast to neuropeptidergic melanocortin neurons, the receptors *MC4R* and *MC3R* are expressed more diffusely (Extended Data Fig. 7a–d). We looked at clusters in the 95th percentile of *MC3R* and *MC4R* expression and examined the concordance between human and mouse (Extended Data Fig. 7e and Supplementary Table 15). We detected several PVN clusters that express *MC4R* as well as *TRH* and to a lesser extent *CRH* (C4-293, C4-296 and C4-315), which likely receive inputs from ARC POMC and AgRP neurons. Furthermore, we observe high *MC4R* expression in a cholinergic cluster (C4-194) mapping to the LPOA and two *HMX3*-expressing clusters (C4-144 and C4-154) probably located in the medial preoptic area and lateral hypothalamus, respectively. Although corresponding mouse cell types for these three clusters exist, they do not express *Mc4r*.

Previously, we have shown that *MC3R* is expressed by ARC GHRH and KISS1 neurons in mice and humans^5^. Here, *MC3R* expression is detectable in one GHRH (C4-161) and two KISS1 (C4-390, C4-391) clusters (Extended Data Fig. 7e). GHRH neurons are conserved across species and express *Mc3r* in the mouse HypoMap. Although the ARC KISS1 clusters clearly exist in the mouse HypoMap, cluster correlation between humans and mice is low. Spatial mapping of *MC3R*^+^ GHRH (C4-161) and KISS1 neurons shows heterogenous distribution within the ARC (Extended Data Fig. 7f). Other notable *MC3R*-expressing clusters include periventricular *NR5A2*- and *SATB2*-expressing neurons (C4-64), which are conserved between species, as well as VMH neurons (C4-345).

## Human hypothalamic incretin-ome

**Extended Data Fig. 8:**
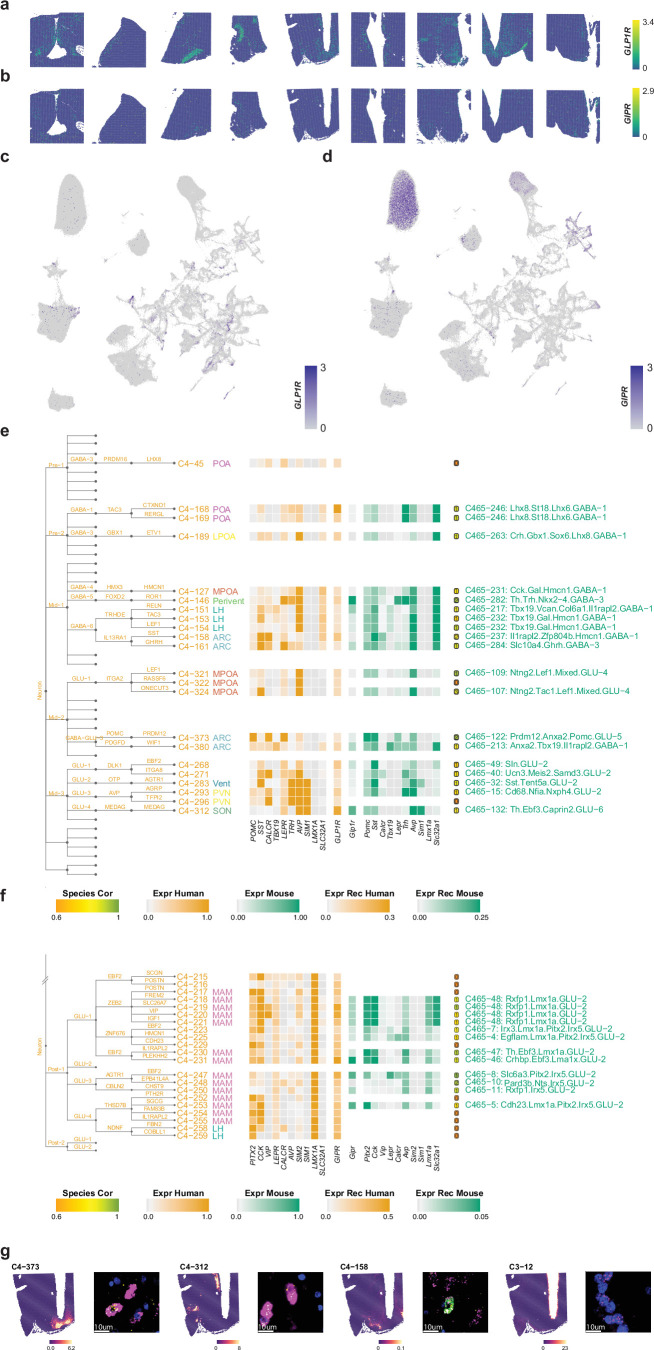
Incretin receptor expression and cross-species comparison. **a-b**, Log-normalized spatial expression of *GLP1R* (**a**) and *GIPR* (**b**) in the human hypothalamus ST sections. **c-d**, UMAP expression plots (log-normalized) showing *GLP1R* (**c**) and *GIPR* (**d**) in the snRNA-seq dataset. **e**, Clusters expressing *GLP1R* in either human or mouse. See Extended Data Fig. 7e for a detailed description **f**, Clusters with *GIPR* expression in the human snRNA-seq data. See Extended Data Fig. 7e for a detailed description. **g**, Spatial mapping of 3 GLP1R clusters (C4-373, C4-312 and C4-158) and 1 GIPR cluster (C3-12) in a medial section of the human hypothalamus. smFISH in the human hypothalamus of near adjacent sections to the medial ST section. From left to right: (1) *GLP1R* (yellow) and *POMC* (pink) co-expression in the ARC. (2) *GLP1R* (yellow) and *AVP* (pink) co-expression in the SON. (3) *GLP1R* (yellow), *CALCR* (pink) and *SST* (green) in the lateral ARC. (4) *GIPR* (pink) in ependymal cells lining the third ventricle. Scale bar = 10 um; n = 2 for each smFISH combination.

Next, we turned our attention to the receptors of the incretin hormones GLP-1 and GIP (Extended Data Fig. 8a–d), with both GLP1R and GIPR being targets for type 2 diabetes mellitus and obesity therapeutics^24^. The 95th percentile of *GLP1R* expression comprises 22 neuronal clusters, 4 of which (C4-312, C4-293, C4-296, C4-283) express *SIM1* and *AVP* (Extended Data Fig. 8e and Supplementary Table 16). C4-312 has the highest expression of *GLP1R*, co-expresses *GIPR* and maps to the PVN and SON (Extended Data Fig. 8e,g). The *SIM1*^+^ clusters C4-293 and C4-296 both map to the PVN. Species conservation of mouse *Sim1p*^+^/*Avp*^+^ cell-type identity and *Glp1r* expression highlights several discordances (Extended Data Fig. 8e).

In humans, the ARC C4-158 cluster expresses *SST*, *GAL*, *CALCR* and *GLP1R*, whereas no *Glp1r* expression is detected in its corresponding mouse cluster (Extended Data Fig. 8e). On the contrary, the *Sst-* and *Glp1r* co-expressing clusters from mouse are conserved in human but do not express *GLP1R* themselves (C4-359). The human C4-158 neurons spatially map to the MBH, but lateral to the ARC (Extended Data Fig. 8g). The second highest expression of *Glp1r* in the mouse HypoMap was found in the cluster ‘C465-282, Th.Trh.Nkx2-4.GABA-3’, identified previously to express both *Glp1r* and *Lepr*, and to play a role in food intake suppression^25^ and preingestive satiation^26^. In humans, we find a highly correlated cluster, C4-146, which also co-expresses *LEPR* and *GLP1R* (Extended Data Fig. 8e).

*GIPR*, unlike *GLP1R*, is expressed in both neurons and non-neurons. Neuronal *GIPR* is identified predominantly in *LMX1A-*expressing posterior hypothalamic subclusters (Extended Data Fig. 8b,d,f). We also detect high *GIPR* expression in ependymal cells (C3-12) surrounding the third ventricle (Extended Data Fig. 8g). Collectively, these data provide a high-resolution expression pattern of incretin hormone receptors in both neuronal and non-neuronal cells in the human hypothalamus.

## BMI GWAS at cellular resolution

**Fig. 5:**
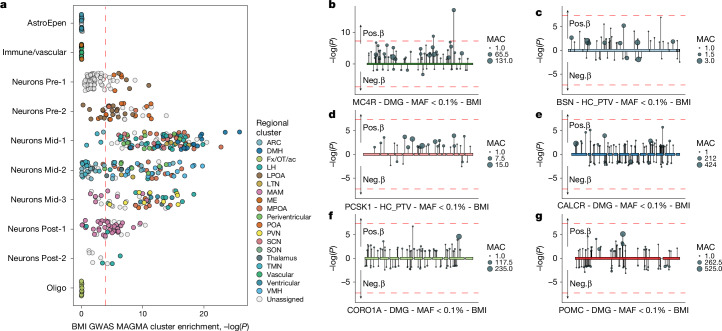
Neuronal clusters are enriched for genes linked to BMI variation in the general population. **a**, Prioritization of 452 human hypothalamic cell types (C4) identified 291 cell types as significantly enriched for associations in the BMI GWAS. Cell types were grouped by cluster level C1 (neurons) and C0 (non-neurons). Dashed line represents the Bonferroni significance threshold, *P* < 0.00011 (two-tailed). Clusters are coloured based on their assigned regions, as seen in Fig. 3. Extended data are shown in Supplementary Table 17. **b**–**g**, Variant-level associations in identified effector genes in UK Biobank. Rare exome variant associations with BMI for variants within *BSN* (**b**), *CALCR* (**c**), *CORO1A* (**d**), *MC4R* (**e**), *PCSK1* (**f**) and *POMC* (**g**). Variant collapsing masks included variants with a MAF < 0.1% and annotated as either high-confidence PTV (HC_PTV) or HC_PTV plus missense variants with a high CADD score (greater than or equal to 25, denoted DMG). Each variant association is represented by a circle and vertical line: line length, *P* value (−log_10_), in the direction of its effect on BMI in carriers of the rare allele; circle size, number of carriers of each variant (allele count). Exons are indicated by boxes and connected by the intron line. Extended data, including individual *P* values, are shown in Supplementary Tables 18 and 19.

Finally, we asked which hypothalamic cell types are implicated in the genetic regulation of obesity. We first integrated HYPOMAP with data from a common variant GWAS of BMI in up to 806,834 people^27^, using CELL-type Expression-specific integration for Complex Traits (CELLECT)^28^ and Multi-marker Analysis of GenoMic Annotation (MAGMA)^29^. We find that 291 out of the 452 hypothalamic cell types at level C4 show significant enrichment of BMI GWAS signals (at Bonferroni corrected *P* < 0.05/452; Fig. 5a and Supplementary Table 17).

Most enriched cell types are mid-hypothalamus neurons, especially C1-6 Mid-1 with 102 clusters (96% of all C4 clusters), C1-10 Mid-2 with 58 clusters (64%) and C1-9 Mid-3 with 48 clusters (86%). No non-neuronal and only few neuronal clusters in C1-5 Pre-1 (15, 25%) and C1-11 Post-2 (3, 33%) showed enrichment. The most significantly enriched neuronal cluster C4-77 (*P* = 1.43 × 10^−26^) is *PTGFR*- and *ONECUT1/2/3*-expressing and maps to the DMH. Multiple *HMX3*- and *NPSR1*- (C4-121, C4-130) or *GAL*- (C4-138) expressing clusters from the medial preoptic area (C4-121) and lateral hypothalamus (C4-138) are strongly enriched as well. Additionally, cluster C4-333, which maps to the lateral VMH and is marked by *FEZF1*, *NTNG1* and *FAM9B*, was highly enriched in the BMI GWAS (sixth, *P* = 1.65 × 10^−20^). Among the other significantly enriched neuronal populations, we find multiple clusters of *SST*-expressing neurons located in or close to the LTN, which have been implicated previously in feeding^30^. This includes a population expressing *P2RX2* in a highly specific manner, C4-361 (*P* = 4.19 × 10^−9^), as well as multiple clusters co-expressing *SST* and *CALCR*, such as the previously discussed *GLP1R*-expressing cluster C4-158 (*P* = 7.57 × 10^−14^).

We next sought to identify ‘effector’ genes that might be driving these associations, defined as over the 95th percentile for cell-type specificity and in the top 1,000 MAGMA gene associations derived from the GWAS data (using CELLECT-GENES). This yielded 426 genes (Supplementary Table 18), most of which (386 of 426) were identified as effector genes in neurons and in the BMI GWAS enriched neuronal subpopulations (396 of 426).

To determine whether disruption of these effector genes influences obesity risk at the population level, we used exome-sequencing data from the UK Biobank study (*n* = 419,692)^31^. We performed rare variant burden tests towards BMI for variants with a minor allele frequency (MAF) less than 0.1% that are either protein truncating variants (PTVs) or missense variants with a high CADD score (greater than or equal to 25; Methods). We find that carrying rare deleterious variants in six (of 426) effector genes is associated significantly with changes in BMI (*P* < 0.05/426; Fig. 5b–g and Supplementary Table 19). Reassuringly, these include well-established causes of monogenic obesity and previously reported associations; *MC4R*^32–35^, *PCSK1* (refs. ^32,36^), *POMC*^37^ and *CALCR*^32^. However, our analyses also highlighted two new genes: *BSN*, encoding a presynaptic protein with a role in exocytosis-mediated neurotransmitter release^38^, which we have shown recently is associated with increased risk of severe obesity, metabolic dysfunction-associated steatotic liver disease and type 2 diabetes^39^; and *CORO1A* (*n* = 415 carriers, *β* = 0.98 ± 0.215; *P* = 5.6 × 10^−6^), which encodes a WD repeat protein involved in cell cycle progression, signal transduction, apoptosis and gene regulation^40^—a gene previously unlinked with obesity.

## Discussion

In hypothalamic research, the vast majority of ‘ground truths’ have, until recently, emerged from mouse neuroanatomical and functional studies. The maturation of single-cell technologies has ushered in a new era of possibilities in human brain mapping. Whereas ‘whole-brain’ single-cell datasets are emerging from developing^41^ and adult^12^ humans, here we provide a detailed, high-resolution spatio-cellular map of the adult human hypothalamus.

It is the spatial element that provides a rich and new dimension to the increasingly ubiquitous single-cell data. Often neglected non-neuronal cell types serve as prime examples. The snRNA-seq data identifies three astrocyte and two oligodendrocyte clusters at C2 that show previously unappreciated spatial segregation (Extended Data Fig. 4). Another advantage of combining single-cell and spatial data is the emerging synergy of both technologies. Because brain banks typically hemisected brains for banking, the median eminence tends not to be sufficiently captured. Thus, tanycytes, which are enriched in the median eminence, are often underrepresented in snRNA-seq data, with only around 100 cells identified here. Yet, the expression profile of the tanycytes can be mapped clearly onto the spatial whole transcriptome data (Extended Data Fig. 4).

The leptin–melanocortin system represents a central appetitive control pathway, the principal components of which were all uncovered more than 20 years ago using genetics^2,3^, but whose mapping in the human hypothalamus we explore here in detail. We demonstrate spatially distinct populations of POMC (Fig. 4b–d), MC4R and MC3R (Extended Data Fig. 7e) neurons. With up to 0.3% of the general population carrying pathogenic mutations in *MC4R*^42^ and drugs targeting this pathway available, there has never been a more relevant time to increase our understanding about this pathway in the human context.

Comparing human and mouse neuronal clusters, we find good overall concordance of neuronal cell types. We do, nevertheless, identify pertinent differences in conservation of gene expression, key among which is that *GLP1R*/*POMC/LEPR* are almost exclusively co-expressed in humans, whereas *Glp1r*- and *Lepr*-expressing POMC neurons are two distinct populations in mice.

The receptors of the moment, however, and of broadest societal relevance, are the incretin hormone receptors, GLP1R and GIPR—both key targets for anti-obesity therapy development^24^. Here we confirm that human hypothalamic *GLP1R* expression is principally neuronal, and catalogue human *GLP1R*^+^ cell types, including one ARC *POMC*^*+*^ cluster and several PVN/SON *AVP*^+^ clusters. In addition to differences in the *GLP1R*/*POMC/LEPR* co-expression, we find that, although SST neurons express *GLP1R* in both humans and mice, their cell-type identity is distinct. It is also interesting that we find a *GLP1R*^+^/*CALCR*^+^ cluster that is spatially distinct, given the efficacy of combining semaglutide with the amylin analogue cagrilintide for weight loss^43^.

In contrast, *GIPR* is expressed in both non-neurons and neurons, consistent with our previous observations in the mouse^14^ and human hypothalamus^44^, and the mouse hindbrain^45^. The *GIPR*^+^ population that most intrigued us are the ependymal cells lining the third ventricle (Extended Data Fig. 8g). Heterozygous loss of function mutations in *GIPR* are associated with lower BMI^46^, whereas pharmacological studies in humans indicate that both agonism and antagonism of this receptor can augment weight loss^47,48^. Could this spatial localization of *GIPR* to the ependymal layer be evidence that, as with tanycytes^49^, they regulate the transport of hormones and key metabolites in and out of the hypothalamus? Although further work will be required to address these questions, our data illuminating the high-resolution expression profile of hypothalamic incretin receptors in a human context are an important first step.

Viewing HYPOMAP through a genetics lens, we find significant expression enrichment of BMI-associated genes in neurons, which is coherent with our current understanding that the large variation in bodyweight is driven primarily by neuronal mechanisms. That *SST*-, *CALCR*- and *GLP1R*-expressing neurons are enriched in the BMI GWAS echoes their enrichment in mouse hindbrain *Calcr*^+^/*Glp1r*^+^ neurons^10^. Finally, gene burden analysis of the 426 ‘effector’ genes that drove the enrichment further corroborates six genes in which rare deleterious variants were associated significantly with changes in BMI, four of which, *MC4R*, *PCSK1*, *POMC* and *CALCR*, having well-established links to BMI. It is gratifying that our approach also highlighted *BSN*, a gene we have shown recently to be linked to obesity^39^, and *CORO1A*, an entirely new player in the regulation of energy balance, thus highlighting HYPOMAP as a platform for discovery.

There are, of course, limitations to our study. First, it is crucial to remember that transcriptomic data, of all types, are designed to identify what is expressed, as opposed to what is not. Second, HYPOMAP has been derived from relatively few donors (11 for the snRNA-seq dataset and 7 for the spatial data, imbalanced in terms of sex), limiting us from deep quantitative analyses, such as effectively comparing differences between the male and female brain. Third, our snRNA-seq donors were of normal weight when they died, so, although of interest in and of itself, the long-term value of these data, given the role of the hypothalamus in maintaining homeostasis, is as a baseline to study this brain region in states of disrupted homeostasis. This will require the difficult and long-term prospective recruitment of donors suffering from relevant diseases, in our case, severe obesity.

Finally, HYPOMAP is not meant to be a static resource. The HYPOMAP framework is designed to be built upon and modified easily. Thus, as data generated from new single-cell spatial approaches emerge, these can be integrated, allowing HYPOMAP to evolve continually. New developments in higher resolution spatial transcriptomics^50^ will certainly also help to further increase spatial resolution. Given our field of expertise, our initial focus has naturally been the appetitive control circuitry. Clearly, this barely begins to scratch the surface of possibilities with this dataset. We hope that by making HYPOMAP open access it will help illuminate human relevant neuronal populations and circuits more broadly, thus enabling the identification of new druggable targets for treating a wide range of conditions linked to the hypothalamus.

## Methods

### Human post-mortem sample preparation

Anonymized human samples were obtained from The Edinburgh Brain and Tissue Bank, MRC London Brain Bank for Neurodegenerative Diseases, Cambridge Brain Bank, South West Dementia Brain Bank, Parkinson’s UK Brain Bank and University of Leipzig Medical Centre Institute of Anatomy, in line with each bank’s Research Ethics Committee approval. Subjects were approached in life for written consent for brain banking, and all tissue donations were collected and stored following legal and ethical guidelines. Donor details for snRNA-seq, spatial transcriptomics and smFISH are given in Supplementary Table 1.

For snRNA-seq, frozen blocks of post-mortem hypothalamus were sourced from adult donors with BMI ranging from 18 to 28 kg m^−2^ and no significant neuropathology. Dissections were performed following delineation of relevant anatomy in cresyl-violet-stained sections from the anterior and posterior surfaces of each sample by a consultant pathologist. Samples from the relevant region were then acquired using a punch biopsy or macrodissected from 100-μm-thick frozen cryostat sections spanning the whole specimen.

For spatial transcriptomics, post-mortem formaldehyde-fixed, paraffin-embedded (FFPE) human brain samples covering the hypothalamus were obtained from the MRC Brain Bank Network. Selection of samples and areas to include in spatial transcriptomics analyses were based on anatomical landmarks using Luxol fast blue/haematoxylin-eosin staining of myelinated fibres and cell bodies; *n* = 9 samples from *n* = 7 different donors (2 male, 5 female). BMI ranged from 16 to 41 kg m^−2^ at the time of death.

### Nucleus dissociation and RNA sequencing

Nuclei were isolated by Dounce homogenization and purified using a protocol modified from ref. ^14^. Briefly, chopped samples were transferred to a 15-ml Dounce homogenizer with 5 ml homogenization buffer (100 μM of dithiothreitol (Sigma–Aldrich), 0.1% Triton X-100 (Sigma–Aldrich), 2× EDTA Protease Inhibitor (Roche), 0.4 U μl^−1^ RNasin RNase inhibitor (Promega; 10,000 U, 40 U ml^−1^) and 0.2 U μl^−1^ Superase.In RNase Inhibitor (Ambion; 10,000 U, 20 U μl^−1^) in nuclei isolation medium (250 mM sucrose, 25 mM KCl (Ambion), 5 mM MgCl_2_ (Ambion) and 10 mM Tris buffer, pH 7.0 (Ambion) in nuclease-free water (Ambion)) with 1 μl ml^−1^ DRAQ5 (Biostatus), and dissociated mechanically using 10 strokes with pestle A and 20 strokes with pestle B. Homogenates were filtered through a 100-μm filter and centrifuged at 600*g* for 5 min in a precooled centrifuge. The supernatant was discarded and the pellet resuspended in 27% Optiprep solution diluted in homogenization buffer and centrifuged at 13,600*g* for 20 min at 4 °C. The nuclear pellet was collected and resuspended in wash buffer (1% BSA, 0.4 U μl^−1^ RNasin and 0.2 U μl^−1^ Superase.In in PBS (Sigma–Aldrich)) and centrifuged at 700*g* for 5 min at 4 °C. This was repeated twice before being passed through a 40-μm cell strainer and this final sample was used to create sequencing libraries. For two donors, single nuclear suspensions were sorted using fluorescent-activated nucleus-sorting (FANS) on a BD FACSMelody instrument. The gating was set according to forward scatter, side scatter and fluorescence at 647/670 nm to detect DraQ5 nuclear staining, and 567 nm to detect NeuN-PE staining. NeuN^+^ events were sorted into a collection tube to enrich for neuronal nuclei.

Sequencing libraries were generated using 10x Genomics Chromium Single-Cell 3′ Reagent kits (v.3.1) according to the standardized protocol. cDNA was amplified for 19 cycles. Paired-end sequencing was performed using an Illumina NovaSeq 6000.

### Sequence alignment, cell calling and quality control

Raw sequence reads were mapped and genes counted based on the Human GRCh38, Ensembl 98 gene model, both using 10x Genomics CellRanger v.4-5 (https://support.10xgenomics.com/single-cell-gene-expression/software/pipelines/latest/what-is-cell-ranger) using the parameter --include-introns. CellBender v.2.0 (ref. ^51^) was used to recalibrate unique molecular identifier (UMI) counts and cell calling.

After removal of flagged nuclei, our snRNA-seq dataset included 571,091 nuclei from 58 samples, which contributed between 748 and 45,771 cells. We used scran’s quickCluster function^52^ to obtain an initial set of clusters that were used as input cluster assignments to scDblFinder, which was run with multiSampleMode set to ‘split’^53^. We additionally ran an initial Seurat-based processing of the whole dataset, including detection of highly variable features, scaling of data, principal component analysis and preliminary clustering^54^. All nuclei detected by scDblFinder as doublets or that were part of Seurat clusters with more than 75% of doublets were removed. We further filtered the data using the sample-based thresholds and additionally set a global threshold of maximum mitochondrial RNA of 10% and a minimum of 800 UMIs per nucleus. After filtering the dataset for doublets and low-quality nuclei, it comprised 353,678 nuclei from the 58 samples, which contributed between 609 and 20,424 nuclei.

The processed snRNA-seq data of all hypothalamus samples (ROIGroupCoarse = ‘Hypothalamus’) were extracted from the loom file published by Siletti et al.^12^. This included a total of 134,471 nuclei that we merged with data from our own study.

### snRNA-seq integration

Our combined human dataset includes 82 10x samples from 11 different donors and two independent studies with a total of 488,149 cells after merging and initial quality control. To integrate all cells and make the data comparable we used scvi-tools (v.0.19.0)^55^, which we have shown previously to be a powerful integration tool that preserves cell-type purity while removing batch differences^14^; scvi always models the library size (nUMI) and we used the sample ID as the covariate (‘batch_key’) to allow future use with scArches. Similar to our previous study we optimized the main hyperparameters of scvi by running a grid search over pre-defined parameter ranges using our published pipeline (https://github.com/lsteuernagel/scIntegration). scIntegration evaluates different scvi model outputs for mixing of samples (using the entropy of the sample distribution in each cell’s nearest neighbours), the purity of cells (cell-type distribution in each cell’s nearest neighbours) and the average silhouette width for cluster separation. We defined a set of ground truth cell types using signatures for mouse glial cell types from our mouse HypoMap^14^ and additionally added a set of manually curated neuron signatures (Supplementary Table 3). We then visualized the hyperparameters of all runs by the evaluation metrics to choose a final set of optimal parameters. Overall, all models integrated the data well and we mostly found small improvements (Supplementary Table 4). The final scvi model was trained for 100 epochs with a dropout rate of 0.1. The model had two layers and 256 nodes per layer (*n* hidden) and the latent space had 80 dimensions. All other parameters were set to default.

### snRNA-seq clustering and annotation

The integrated embedding from the final scvi model was used for downstream analysis. We adapted our previous dataset harmonization pipeline^14^ for many of the following steps but changed it where necessary. We started with an initial round of clustering and annotated these clusters using marker gene signatures for principal cell types, including some non-hypothalamic ones. We found several clusters of cells that probably reside outside the hypothalamus (for example, *SCL17A7*^+^ neurons or thalamic *SHOX2*^+^ neurons). After annotating all cells, we removed the likely non-hypothalamic clusters and a few clusters representing low-quality cells, leaving us with a final dataset of 433,369 cells. Due to the imbalance of main cell-type distribution (for example, 40.4% of all cells are oligodendrocytes), we split the data into four main subsets for clustering and tree building: neurons, Oligo, AstroEpen and other non-neuronal cells. We ran Leiden clustering on different resolutions 100 times and combined them into a single consensus clustering per resolution using hybrid bipartite graph formulation^56^ to improve robustness. For each subset, several flat consensus clusters were combined into a consensus hierarchical tree using mrtree^57^. Marker genes of each cluster versus all others, as well as only its sibling nodes in the subtree were calculated using a batch-stratified Wilcoxon rank sum test^58^ and corrected for multiple testing using Bonferroni correction. The subtrees were pruned by merging nodes with insufficient differences (fewer than five strong marker genes, fewer than 50 cells or more than 90% of cells originating from a single donor) with their closest sibling node based on Euclidean distance in the integrated embedding. We repeated this pruning five times and used the final hierarchical tree in the following step. We then merged all four subtrees into the final clustering tree, which spans five distinct levels (C0–C4) with 4–452 distinct clusters; however, for non-neuronal cell types only up to four levels exist^14^. We manually labelled the first levels of the tree (C0, C1) based on cell type (broad class) and general location for neurons. For glial cells, we additionally annotated clusters with common names on levels C2 and C3 where applicable. For neurons, on level C2, we used neurotransmitter identity and consecutive numbers to label clusters. On levels C3 and C4 we used up to two marker genes to label clusters. Marker genes with high specificity both versus all other clusters and versus sibling clusters were prioritized. For four clusters of AgRP, NPW, HDC and PMCH neurons, we manually overwrote the label since the key neurotransmitter genes were not the top-scoring gene. When analysing genes of interest, we used the 99th (*POMC*, *AGRP*) or 95th (receptors) percentile of expression percentage as cutoff to select a subset of clusters for detailed examination.

### Cross-species comparison

The cross-species integration with the mouse HypoMap dataset^14^ was conducted using only the neurons from both species. An overview of the pipeline can be found in Extended Data Fig. 5a. Homologous genes were identified using Ensembl v.101 (ref. ^59^), corresponding to Gencode v.35 used by Siletti et al.^12^. To reduce 1:*N* gene relationships, only the gene with the highest sequence homology was retained. The remaining 18,279 homologous genes were used to subset the expression matrices for both species. Highly variable genes (HVGs) were selected for each species individually, by identifying HVGs per sample (human) or batch (mouse) and ranking by occurrence. A total of 2,500 HVGs were selected per species and the overlap of 1,404 genes was used as input to an scvi model to obtain an integrated embedding including both species. The parameters for scvi were adapted from the HYPOMAP scvi model described above. To achieve more aggressive mixing and move cells from the two species closer together, the number of training rounds (epochs) was increased to 600.

Cluster averages of the scvi embedding were calculated for clusters C4 in human and C465 in mouse. The Pearson correlation coefficients of cluster averages between species were used to identify corresponding (‘matched’) clusters between species. To remove *M*:*N* relationships, the correlations were adjusted and filtered: first, we grouped by either human or mouse cluster and obtained the maximum correlation value for each cluster (human and mouse). Then, for all correlation values of each cluster, the difference between the actual values and the maximum correlation was subtracted from the actual correlation values to obtain an adjusted value. Next, a graph was constructed with clusters as nodes and edges between all clusters across species with an adjusted correlation greater than 0.7. To remove all remaining *M*:*N* relationships the graph was pruned so that, for any node, all 1:*N* edges were kept if the neighbouring clusters had no edges to other nodes. If neighbouring nodes had several edges, only the edge with maximum adjusted correlation was retained.

Uniprot^60^ was queried using the REST API to obtain a list of reviewed GPCRs for both species, which was merged and used to select the most specific receptors in clusters of interest. For *AGTR1* we included only mouse *Agtr1a* in the figure because *Agtr1b* was not expressed in mouse. We also excluded *Npy2r*, which was nearly absent in the human snRNA-seq data but detected robustly in the spatial transcriptomic data of the hypothalamus.

### 10x Genomics Visium CytAssist spatial transcriptomics

FFPE sections (5 μm) were prepared using a microtome (Leica) in an RNase-free environment and mounted onto positively charged slides. The sections were then stored at room temperature until use. Slides were processed for spatial transcriptomics according to 10x Genomics Visium CytAssist v.2 protocols. Briefly, samples were deparaffinized in xylene and a series of concentrations of ethanol solutions (100% to 70%) and immersed in water before haematoxylin and eosin staining. Once stained, samples were cover-slipped using a glycerol mountant and imaged using a VS200 slide scanner (Olympus Life Science) at ×20 magnification (air objective, 0.8 numerical aperture). Coverslips were removed and samples underwent destaining and decrosslinking, and were incubated overnight with 10x Genomics Visium Human WT Probes v.2 (Pleasanton). Following this, slides were loaded at the appropriate orientation, along with the Visium 11 × 11-mm gene expression slide, onto a CytAssist (10x Genomics), where hybridized probes were released from the tissue and ligated to spatially barcoded oligonucleotides on the Visium Gene expression slide. A tissue image was taken on the CytAssist at ×10 magnification for downstream alignment of library to the tissue section. Barcoded ligation products were then amplified to create a cDNA library for sequencing.

Libraries from the nine samples were pooled and sequenced on a NovaSeq 6000 sequencing platform (Illumina), using a NovaSeq 6000 S2 Reagent Kit v.1.5 (Illumina) according to the manufacturer’s instructions. Subsequently, fastq files were generated for each sample, reads were aligned to their corresponding probe-sequences (Visium human transcriptome probe set v.2, based on GRCh38 2020-A), mapped back to the Visium spot where a given probe was originally captured and finally aligned to the original HE-stained image of the tissue section using SpaceRanger v.2.0.0 (10x Genomics).

Atlas location of each spatial transcriptomics section was determined by consulting the Atlas of the Human Brain (4th edn)^61^ (Supplementary Table 10).

### Spatial transcriptomics data analysis

Across the nine samples, the median number of counts per Visium spot was 7,105, and the median number of detected genes per spot was 3,560. The average sequencing saturation was 0.68. Furthermore, for each individual sample, graphs with (1) sequencing saturation and (2) detected number of genes plotted as a function of median number of reads per spot revealed the plateau phase was either obtained or clearly approached, that is, very little benefit would be gained from even deeper sequencing.

### Spatial transcriptomics data pre-processing

The number of genes per spot and counts per spot was inspected for each tissue section individually using the Loupe browser to identify whether there were areas of the sample that had unusually low/high counts that are probably artefacts from the experimental procedures. These spots were identified and removed from downstream analysis.

For visualization of gene expression in the spatial transcriptomics data, data were analysed using Seurat (v. 4.3.0)^62^. Raw count matrices along with spatial barcode coordinates for each sample were loaded, and data was log-normalized for visualization of transcript expression.

### Integration of snRNA-seq and spatial transcriptomic data: cell2location

We used cell2location (v.0.1.2)^18^ to predict the locations of snRNA-seq cell populations in the spatial transcriptomics data. We utilized the entire snRNA-seq dataset as a reference, and estimated reference cell-type signatures for clustering levels C1–C4. We included genes that were expressed in at least 8% of cells, and genes expressed in at least 0.05% of cells if the non-zero mean was greater than 1.4. We estimated reference signatures using the negative binomial regression model, accounting for the effects of donor, sex, batch and dataset.

For each cluster level, we trained the cell2location model with a detection *α* of 20 and three cells per location as hyperparameters, and trained for 30,000 epochs, with the final gene list including genes expressed in both the snRNA-seq and spatial transcriptomics dataset. Results were visualized using scanpy and Seurat. The plots represent the estimated abundance of cell types at each location.

To cluster the spatial transcriptomics spots, we used *k*-nearest neighbours and Leiden clustering on a matrix of cell abundance scores for each C3 neuronal snRNA-seq cluster and C2 non-neuronal snRNA-seq cluster. We used the C3 neuronal and C2 non-neuronal abundance mappings as these levels provided greater number of clusters mapping confidently to regions in the spatial transcriptomics dataset. We annotated each cluster based on the hypothalamic region in which most spots were present, and by the top marker genes for each cluster. If several spatial transcriptomics clusters originated from the same hypothalamic region, then these were grouped together for regional annotation of the spatial transcriptomics dataset.

### Assigning regional annotations to snRNA-seq clusters

To assign snRNA-seq clusters to spatial transcriptomics regional clusters, we identified the (ungrouped) region in which the adjusted mean abundance score (median regional abundance subtracted from the mean abundance score for a snRNA-seq cluster in a region) for each C3 neuronal cluster and C2 non-neuronal cluster was the highest. We then calculated the median absolute deviation (MAD) for each cluster in each spatial region (ungrouped) and normalized the adjusted abundance for each snRNA-seq cluster in each region by dividing it by the MAD (we call this ‘mad_x’). If the region with the highest adjusted mean abundance score for a particular cluster also had a mad_x > 10, then this region was assigned to this cluster. A mad_x < 10 indicated low confidence mapping to any region and these snRNA-seq clusters were not assigned to a regional cluster. The regional annotation for some clusters were adjusted manually if the regional assignment did not match biology (for example, some clusters mapping to the LTN were generally thought to be anterior or pre-hypothalamus and so were manually assigned ‘NA’), or if mad_x < 10 but the cluster showed good abundance in the appropriate region. Overall, we found the C3 neuronal and C2 non-neuronal abundance estimates to be very robust and therefore assigned C4 snRNA-seq clusters to regional clusters by using their C3 parent’s assignment. We used C3-propogated assignments to generally label all C4 clusters, but showed C4 abundances in some specific cases. An overview of the region assignments can be found in Supplementary Table 12. The mean cell abundance score for C3 and C4 clusters can be found in Supplementary Tables 11 and 13, respectively.

### Software and packages used for snRNA-seq and spatial transcriptomics analysis

The following R and Python packages were used for the analysis and plotting of snRNA-seq and spatial transcriptomics datasets: Python v.3.10.8–v.3.10.12, scvi v.0.19.0, scanpy v.1.9.8, pandas v.1.4.4, numpy v.1.26.4, cell2location v.0.1.2, cellbender v.0.1–v.0.2, cellex v.1.2.2, CELLECT v.1.3.0, R v.4.3.1, future.apply v.1.11.1-9001, future v.1.33.1-9009, pbapply v.1.7-2, Matrix v.1.6-1.1, scUtils v.0.0.1, magrittr v.2.0.3, igraph v.1.5.1, treeio v.1.26.0, ggh4x v.0.2.6, scales v.1.2.1, edgeR v.4.0.16, limma v.3.58.1, ggtree v.3.10.1, lubridate v.1.9.3, forcats v.1.0.0, stringr v.1.5.0, dplyr v.1.1.3, purrr v.1.0.2, readr v.2.1.4, tidyr v.1.3.0, tibble v.3.2.1, ggplot2 v.3.4.4, tidyverse v.2.0.0, SeuratObject v.4.1.4, Seurat v.4.4.0, RcppAnnoy v.0.0.22, cellranger v.4-5, spaceranger v.2 and bolt-lmm v.2.3.6.

### Single-molecule fluorescence in situ hybridization

FFPE sections (5 μm) from the same tissue blocks used for spatial transcriptomics (see Supplementary Table 1 for donor information) were cut and mounted onto positively charged slides. Multiplex fluorescence RNAScope (ACDBio) was performed using a Bond RX fully automated research stainer (Leica), the RNAScope LS multiplex fluorescent reagent kit (Advanced Cell Diagnostics (ACD), Bio-Techne) and probes specific for *GLP1R* (catalogue no. 519828), *GIPR* (catalogue no. 471348), *SST* (catalogue no. 310598), *POMC* (catalogue no. 429908) and *AVP* (catalogue no. 401368; Advanced CellH Diagnostics, Bio-Techne). Slides were baked and deparaffinized before heat-induced epitope retrieval at 95 °C for 30 min using Bond ER Solution 2. Next ACD enzyme (ACDBio) was added, and slides were incubated at 40 °C for 15 min. Samples were hybridized, amplified and detected according to the ACD Multiplex Protocol P1. Final detection was achieved with the Opal 570 and Opal 690 fluorophore reagent packs (Akoya BioSciences, Inc., diluted 1:1,000), and samples were counterstained with 4′,6-diamidino-2-phenylindole (ACD) to mark cell nuclei and cover-slipped with ProLong Diamond antifade mountant (ThermoFisher Scientific) before being imaged using the VS200 slide scanner (Olympus Life Science) at ×20 magnification (air objective, 0.8 numerical aperture).

Three independent human samples (see Supplementary Table 1 for donor information) were used to assess ependymal and tanycyte expression markers. Fresh post-mortem human hypothalamus 2 × 3 × 1-cm blocks (less than 24 h post-mortem) were incubated for 16 h in 10% neutral buffered formalin and then further fixed for 48–72 h in 4% paraformaldehyde. Brain blocks were dehydrated in a series of ethanol treatments (70% (16 h, 2 × 4 h), 80% (16 h, 2 × 4 h), 96% (16 h, 2 × 4 h) and 100% (16 h, 1 × 4 h)). The blocks were then incubated for 3.5 days in xylol, followed by two incubations in fresh paraffin (5 h, 16 h) before placing the blocks into forms. Brain blocks were sliced (5 µm) and mounted on Superfrost (ThermoFisher) glass slides and stored at room temperature.

We performed smFISH on human hypothalamic slices as recommended for FFPE-embedded tissue by the manufacturer (RNAScope Multiplex Fluorescent Reagent Kit v.2 Assay, catalogue no. 323100-USM, ACD). Briefly, slides were incubated for 1 h at 60 °C, followed by two 5-min incubations in xylene at room temperature, and two 2-min incubation steps in 100% ethanol. Slides were air-dried and subjected to target retrieval for 15 min. Protease Plus (ACD) was applied for 25 min at 40 °C. After the pre-treatment, the standard protocol was continued. The following RNAScope probes were used: *DIO2* (catalogue no. 562211), *FZD5* (catalogue no. 414051), *STOML3* (custom made) and *LPAR3* (catalogue no. 428811). For controls, 3-plex positive (catalogue no. 320861) and 3-plex negative (catalogue no. 320871) were used. Probes were detected with Opal fluorophores from Perkin Elmer, Opal 690 (catalogue no. FP1497001KT); Opal 620 (catalogue no. FP1495001KT) and Opal 570 (catalogue no. FP1488001KT) at a dilution of 1:1,000. Images were captured using a Leica TCS confocal microscope, equipped with ×20/0.75 liquid immersion and ×40/1.30 oil objectives, and LasX software. Images of the hypothalamus were captured at the hypothalamus and median eminence areas from the anterior to posterior hypothalamus.

### Cell-type enrichment and BMI associations

Cell-type specificity matrices were generated using CELLEX software v.1.2.2 (ref. ^28^). Due to memory limits, we performed bootstrapping by sampling the HYPOMAP dataset randomly into ten smaller datasets, each containing 100,000 cells. CELLEX was then performed on each of the subsets, and the mean values were taken forward for the subsequent enrichment analysis.

**Extended Data Fig. 9:**
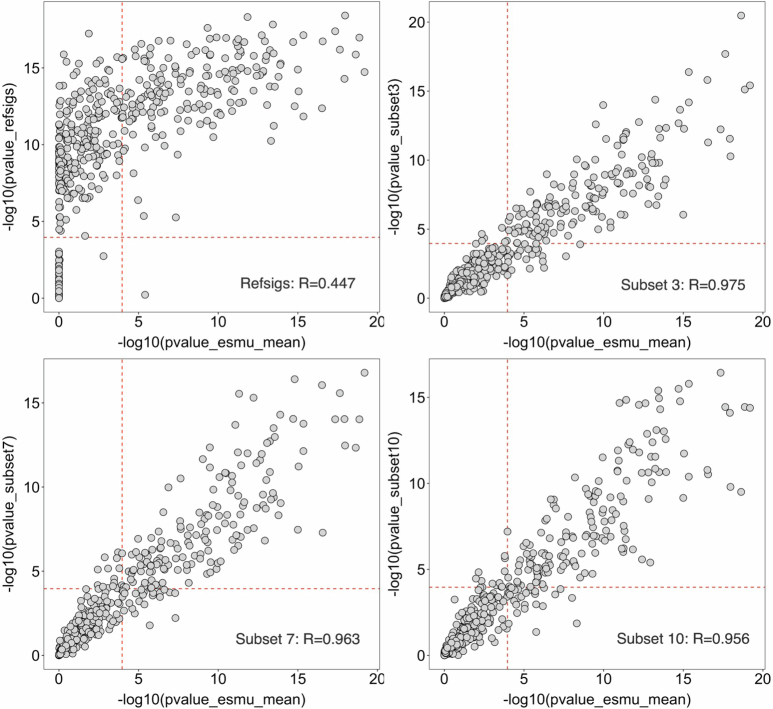
Correlation of MAGMA enrichment across different subsets of cell populations. Cell-type prioritisation across each the 452 human hypothalamic cell types, was compared between three different subsets of 100 K cells and enrichment was calculated using reference signature values generated from cell2location (on the Y axes), and the dataset used in discovery (on the X axes). The Pearson correlation for each comparison is displayed (two-sided).

Using the resulting cell-type specificity matrices, we ran CELLECT^28^ with MAGMA^29^, alongside GWAS data from the GIANT BMI meta-analysis (*N*_max_ = 806,834)^27^, to prioritize hypothalamic cell types that showed enrichment in the BMI GWAS. CELLECT-MAGMA (v.1.3.0) was run with default parameters across the 452 tested hypothalamic cell types, setting the multiple-test corrected significance threshold at *P* < 0.05/452 and followed-up by CELLECT-GENES, but setting the percentile cutoff to 95. CELLECT-MAGMA was also run on reference signature values from cell2location and the above-mentioned subsets as a sensitivity analysis (Extended Data Fig. 9).

We analysed exome-sequencing-based rare variant burden, as described in Gardner et al.^63^ using data from up to 454,787 individuals from the UK Biobank study^31^ through the UK Biobank Research Access Platform (https://ukbiobank.dnanexus.com). Variants were then annotated with the ENSEMBL Variant Effect Predictor (VEP)^64^ v.10448 with the ‘everything’ flag and the LOFTEE plugin^65^ and prioritized a single MANE v.0.97 or VEP canonical ENSEMBL transcript and most damaging consequence as defined by VEP defaults. To define PTVs, we grouped high-confidence (as defined by LOFTEE) stop gained, splice donor/acceptor and frameshift consequences. All variants were subsequently annotated using CADD (v.1.650)^66^. BMI for all participants was obtained from the UK Biobank data showcase (field 21001). After excluding people with missing data, 419,692 people with BMI measures remained for downstream analysis. To assess the association between rare variant burden and BMI, we implemented BOLT-LMM (v.2.3.551)^67^, using a set of dummy genotypes representing the per gene carrier status. For the latter, we collapsed variants with a MAF < 0.1% across each gene and defined carriers of variants as those with a qualifying high-confidence PTV (HC_–_PTV) as defined by VEP and LOFTEE or ‘damaging’ variants (DMG), including missense variants with a CADD score greater than or equal to 25 and the aforementioned HC_–_PTVs. Genes with fewer than ten carriers were excluded. BOLT-LMM was run with default settings and the ‘lmmInfOnly’ flag and all analyses were controlled for sex, age, age2, WES batch and the first ten genetic ancestral principal components as calculated^31^. Gene-level BOLT association summary statistics were then extracted for the 426 identified effector genes, setting the multiple-test corrected threshold at *P* < 0.05/426.

Finally, to identify which GWAS signals were proximal to the identified effector genes, we also performed signal selection on the GIANT BMI GWAS meta-analysis. GWAS summary statistics were filtered to retain variants with a MAF > 0.1% and that were present in at least half the contributing studies. Quasi-independent genome-wide significant (*P* < 5 × 10^−8^) signals were initially selected in 1-Mb windows and secondary signals within these loci were further selected by conditional analysis in GCTA^68^, using a linkage disequilibrium reference derived from the UK Biobank study. Primary signals were then supplemented with unlinked (*R*^2^ < 5%) secondary signals, whose association statistics did not overtly change in the conditional models. Signals were mapped to proximal effector genes, within 500-kb windows. For genes within 500 kb of multiple GWAS signals, the most significant signal is shown in Supplementary Table 19.

Results from CELLECT and exome associations were visualized using ggplot2 (v.3.4.2) in R (v.4.2.1).

### Reporting summary

Further information on research design is available in the Nature Portfolio Reporting Summary linked to this article.

## Online content

Any methods, additional references, Nature Portfolio reporting summaries, source data, extended data, supplementary information, acknowledgements, peer review information; details of author contributions and competing interests; and statements of data and code availability are available at 10.1038/s41586-024-08504-8.

## Supplementary information

Supplementary Information A guide to Supplementary Tables 1–20 (tables supplied separately).

Reporting Summary

Supplementary Tables Supplementary Tables 1–20 and a data dictionary for all supplementary tables with detailed descriptions of the columns in each table.

## Data Availability

The HYPOMAP snRNA-seq data is available in an interactive cellxgene viewer at https://cellxgene.cziscience.com/collections/d0941303-7ce3-4422-9249-cf31eb98c480. Additionally, the Seurat and anndata objects of HYPOMAP (snRNA-seq and spatial transcriptomics) and the scvi model, which are required to reproduce Figs. 1–4, Extended Data Figs. 1–8 and Supplementary Tables 1–16 and to project new data, are deposited at University of Cambridge’s Apollo Repository (10.17863/CAM.111988). The newly generated human snRNA-seq data are deposited at the European Genome-Phenome Archive (https://ega-archive.org/) under accession number EGAD50000000997. The spatial transcriptomics data are available from Gene Expression Omnibus (GEO), accession number GSE278848.

## Code availability

The code used to build HYPOMAP and to create Figs. 1–4, Extended Data Figs. 1–8 and Supplementary Tables 1–16, together with the source data files, can be found at https://github.com/lsteuernagel/HYPOMAP and https://github.com/georgiedowsett/HYPOMAP. The pipeline for evaluation of scvi hyperparameters to optimize the HypoMap model can be found at https://github.com/lsteuernagel/scIntegration. We will provide any other code upon request. Analysis code for the UK Biobank is available at https://github.com/mrcepid-rap.

## References

[1] Fong, H., Zheng, J. & Kurrasch, D. The structural and functional complexity of the integrative hypothalamus. *Science* **382**, 388–394 (2023). 37883552 10.1126/science.adh8488

[2] Loos, R. J. F. & Yeo, G. S. H. The genetics of obesity: from discovery to biology. *Nat. Rev. Genet.* **23**, 120–133 (2022). 34556834 10.1038/s41576-021-00414-z PMC8459824

[3] Yeo, G. S. H. et al. The melanocortin pathway and energy homeostasis: from discovery to obesity therapy. *Mol. Metab.* **48**, 101206 (2021). 33684608 10.1016/j.molmet.2021.101206 PMC8050006

[4] Seminara, S. B. & Topaloglu, A. K. Review of human genetic and clinical studies directly relevant to GnRH signalling. *J. Neuroendocrinol.* **34**, e13080 (2022). 34970798 10.1111/jne.13080 PMC9299506

[5] Lam, B. Y. H. et al. MC3R links nutritional state to childhood growth and the timing of puberty. *Nature* **599**, 436–441 (2021). 34732894 10.1038/s41586-021-04088-9 PMC8819628

[6] Wilding, J. P. H. et al. Once-weekly semaglutide in adults with overweight or obesity. *N. Engl. J. Med.* **384**, 989–1002 (2021). 33567185 10.1056/NEJMoa2032183

[7] Jastreboff, A. M. et al. Tirzepatide once weekly for the treatment of obesity. *N. Engl. J. Med.* **387**, 205–216 (2022). 35658024 10.1056/NEJMoa2206038

[8] Secher, A. et al. The arcuate nucleus mediates GLP-1 receptor agonist liraglutide-dependent weight loss. *J. Clin. Invest.* **124**, 4473–4488 (2014). 25202980 10.1172/JCI75276 PMC4215190

[9] Gabery, S. et al. Semaglutide lowers body weight in rodents via distributed neural pathways. *JCI Insight* **5**, e133429 (2020). 32213703 10.1172/jci.insight.133429 PMC7213778

[10] Ludwig, M. Q. et al. A genetic map of the mouse dorsal vagal complex and its role in obesity. *Nat. Metab.* **3**, 530–545 (2021). 33767443 10.1038/s42255-021-00363-1 PMC12009600

[11] Trapp, C. M. & Censani, M. Setmelanotide: a promising advancement for pediatric patients with rare forms of genetic obesity. *Curr. Opin. Endocrinol. Diabetes Obes.* **30**, 136–140 (2023). 36722447 10.1097/MED.0000000000000798 PMC9973437

[12] Siletti, K. et al. Transcriptomic diversity of cell types across the adult human brain. *Science* **382**, eadd7046 (2023). 37824663 10.1126/science.add7046

[13] Lopez, R., Regier, J., Cole, M. B., Jordan, M. I. & Yosef, N. Deep generative modeling for single-cell transcriptomics. *Nat. Methods* **15**, 1053–1058 (2018). 30504886 10.1038/s41592-018-0229-2 PMC6289068

[14] Steuernagel, L. et al. HypoMap-a unified single-cell gene expression atlas of the murine hypothalamus. *Nat. Metab.* **4**, 1402–1419 (2022). 36266547 10.1038/s42255-022-00657-y PMC9584816

[15] Herb, B. R. et al. Single-cell genomics reveals region-specific developmental trajectories underlying neuronal diversity in the human hypothalamus. *Sci. Adv.* **9**, eadf6251 (2023). 37939194 10.1126/sciadv.adf6251 PMC10631741

[16] Romanov, R. A., Alpar, A., Hokfelt, T. & Harkany, T. Molecular diversity of corticotropin-releasing hormone mRNA-containing neurons in the hypothalamus. *J. Endocrinol.* **232**, R161–R172 (2017). 28057867 10.1530/JOE-16-0256

[17] Hatton, I. A. et al. The human cell count and size distribution. *Proc. Natl Acad. Sci. USA* **120**, e2303077120 (2023). 37722043 10.1073/pnas.2303077120 PMC10523466

[18] Kleshchevnikov, V. et al. Cell2location maps fine-grained cell types in spatial transcriptomics. *Nat. Biotechnol.* **40**, 661–671 (2022). 35027729 10.1038/s41587-021-01139-4

[19] Khodai, T. & Luckman, S. M. Ventromedial nucleus of the hypothalamus neurons under the magnifying glass. *Endocrinology* **162**, bqab141 (2021). 34265067 10.1210/endocr/bqab141 PMC8331052

[20] Endo, F. et al. Molecular basis of astrocyte diversity and morphology across the CNS in health and disease. *Science* **378**, eadc9020 (2022). 36378959 10.1126/science.adc9020 PMC9873482

[21] Campbell, J. N. et al. A molecular census of arcuate hypothalamus and median eminence cell types. *Nat. Neurosci.* **20**, 484–496 (2017). 28166221 10.1038/nn.4495 PMC5323293

[22] Reitman, M. L. et al. Pharmacokinetics and pharmacodynamics of MK-5046, a bombesin receptor subtype-3 (BRS-3) agonist, in healthy patients. *J. Clin. Pharmacol.* **52**, 1306–1316 (2012). 22162541 10.1177/0091270011419854 PMC5137193

[23] Biglari, N. et al. Functionally distinct POMC-expressing neuron subpopulations in hypothalamus revealed by intersectional targeting. *Nat. Neurosci.* **24**, 913–929 (2021). 34002087 10.1038/s41593-021-00854-0 PMC8249241

[24] Drucker, D. J. & Holst, J. J. The expanding incretin universe: from basic biology to clinical translation. *Diabetologia*10.1007/s00125-023-05906-7 (2023). 36976349 10.1007/s00125-023-05906-7

[25] Rupp, A. C. et al. Suppression of food intake by Glp1r/Lepr-coexpressing neurons prevents obesity in mouse models. *J. Clin. Invest.* **133**, e157515 (2023). 37581939 10.1172/JCI157515 PMC10541203

[26] Kim, K. S. et al. GLP-1 increases preingestive satiation via hypothalamic circuits in mice and humans. *Science* **385**, 438–446 (2024). 38935778 10.1126/science.adj2537 PMC11961025

[27] Yengo, L. et al. Meta-analysis of genome-wide association studies for height and body mass index in approximately 700000 individuals of European ancestry. *Hum. Mol. Genet.* **27**, 3641–3649 (2018). 30124842 10.1093/hmg/ddy271 PMC6488973

[28] Timshel, P. N., Thompson, J. J. & Pers, T. H. Genetic mapping of etiologic brain cell types for obesity. *eLife* **9**, e55851 (2020). 32955435 10.7554/eLife.55851 PMC7505664

[29] Zhang, L. et al. A genome-wide association study identified new variants associated with mathematical abilities in Chinese children. *Genes Brain Behav.* **22**, e12843 (2023). 36811322 10.1111/gbb.12843 PMC10067424

[30] Luo, S. X. et al. Regulation of feeding by somatostatin neurons in the tuberal nucleus. *Science* **361**, 76–81 (2018). 29976824 10.1126/science.aar4983

[31] Karczewski, K. J. et al. Systematic single-variant and gene-based association testing of thousands of phenotypes in 394,841 UK Biobank exomes. *Cell Genom.* **2**, 100168 (2022). 36778668 10.1016/j.xgen.2022.100168 PMC9903662

[32] Akbari, P. et al. Sequencing of 640,000 exomes identifies GPR75 variants associated with protection from obesity. *Science* **373**, eabf8683 (2021). 34210852 10.1126/science.abf8683 PMC10275396

[33] Huszar, D. et al. Targeted disruption of the melanocortin-4 receptor results in obesity in mice. *Cell* **88**, 131–141 (1997). 9019399 10.1016/s0092-8674(00)81865-6

[34] Vaisse, C., Clement, K., Guy-Grand, B. & Froguel, P. A frameshift mutation in human MC4R is associated with a dominant form of obesity. *Nat. Genet.* **20**, 113–114 (1998). 9771699 10.1038/2407

[35] Yeo, G. S. et al. A frameshift mutation in MC4R associated with dominantly inherited human obesity. *Nat. Genet.* **20**, 111–112 (1998). 9771698 10.1038/2404

[36] Jackson, R. S. et al. Obesity and impaired prohormone processing associated with mutations in the human prohormone convertase 1 gene. *Nat. Genet.* **16**, 303–306 (1997). 9207799 10.1038/ng0797-303

[37] Krude, H. et al. Severe early-onset obesity, adrenal insufficiency and red hair pigmentation caused by POMC mutations in humans. *Nat. Genet.* **19**, 155–157 (1998). 9620771 10.1038/509

[38] Butz, S., Okamoto, M. & Sudhof, T. C. A tripartite protein complex with the potential to couple synaptic vesicle exocytosis to cell adhesion in brain. *Cell* **94**, 773–782 (1998). 9753324 10.1016/s0092-8674(00)81736-5

[39] Zhao, Y. et al. Protein-truncating variants in BSN are associated with severe adult-onset obesity, type 2 diabetes and fatty liver disease. *Nat. Genet.* **56**, 579–584 (2024). 38575728 10.1038/s41588-024-01694-x PMC11018524

[40] O’Leary, N. A. et al. Reference sequence (RefSeq) database at NCBI: current status, taxonomic expansion, and functional annotation. *Nucleic Acids Res.* **44**, D733–D745 (2016). 26553804 10.1093/nar/gkv1189 PMC4702849

[41] Eze, U. C., Bhaduri, A., Haeussler, M., Nowakowski, T. J. & Kriegstein, A. R. Single-cell atlas of early human brain development highlights heterogeneity of human neuroepithelial cells and early radial glia. *Nat. Neurosci.* **24**, 584–594 (2021). 33723434 10.1038/s41593-020-00794-1 PMC8012207

[42] Wade, K. H. et al. Loss-of-function mutations in the melanocortin 4 receptor in a UK birth cohort. *Nat. Med.* **27**, 1088–1096 (2021). 34045736 10.1038/s41591-021-01349-y PMC7611835

[43] Frias, J. P. et al. Efficacy and safety of co-administered once-weekly cagrilintide 2.4 mg with once-weekly semaglutide 2.4 mg in type 2 diabetes: a multicentre, randomised, double-blind, active-controlled, phase 2 trial. *Lancet* **402**, 720–730 (2023). 37364590 10.1016/S0140-6736(23)01163-7

[44] Adriaenssens, A. E. et al. Glucose-dependent insulinotropic polypeptide receptor-expressing cells in the hypothalamus regulate food intake. *Cell Metab.* **30**, 987–996 e986 (2019). 31447324 10.1016/j.cmet.2019.07.013 PMC6838660

[45] Dowsett, G. K. C. et al. A survey of the mouse hindbrain in the fed and fasted states using single-nucleus RNA sequencing. *Mol. Metab.* **53**, 101240 (2021). 33962048 10.1016/j.molmet.2021.101240 PMC8170503

[46] Turcot, V. et al. Protein-altering variants associated with body mass index implicate pathways that control energy intake and expenditure in obesity. *Nat. Genet.* **50**, 26–41 (2018). 29273807 10.1038/s41588-017-0011-x PMC5945951

[47] Campbell, J. E. Targeting the GIPR for obesity: to agonize or antagonize? Potential mechanisms. *Mol. Metab.* **46**, 101139 (2021). 33290902 10.1016/j.molmet.2020.101139 PMC8085569

[48] Veniant, M. M. et al. A GIPR antagonist conjugated to GLP-1 analogues promotes weight loss with improved metabolic parameters in preclinical and phase 1 settings. *Nat. Metab.* **6**, 290–303 (2024). 38316982 10.1038/s42255-023-00966-w PMC10896721

[49] Prevot, V., Nogueiras, R. & Schwaninger, M. Tanycytes in the infundibular nucleus and median eminence and their role in the blood-brain barrier. *Handb. Clin. Neurol.* **180**, 253–273 (2021). 34225934 10.1016/B978-0-12-820107-7.00016-1

[50] Schott, M. et al. Open-ST: high-resolution spatial transcriptomics in 3D. *Cell* **187**, 3953–3972 e3926 (2024). 38917789 10.1016/j.cell.2024.05.055

[51] Fleming, S. J. et al. Unsupervised removal of systematic background noise from droplet-based single-cell experiments using CellBender. *Nat. Methods* **20**, 1323–1335 (2023). 37550580 10.1038/s41592-023-01943-7

[52] Lun, A. T., Bach, K. & Marioni, J. C. Pooling across cells to normalize single-cell RNA sequencing data with many zero counts. *Genome Biol.* **17**, 75 (2016). 27122128 10.1186/s13059-016-0947-7 PMC4848819

[53] Germain, P. L., Lun, A., Garcia Meixide, C., Macnair, W. & Robinson, M. D. Doublet identification in single-cell sequencing data using scDblFinder. *F1000Res* **10**, 979 (2021). 35814628 10.12688/f1000research.73600.1 PMC9204188

[54] Hao, Y. et al. Integrated analysis of multimodal single-cell data. *Cell* **184**, 3573–3587 e3529 (2021). 34062119 10.1016/j.cell.2021.04.048 PMC8238499

[55] Gayoso, A. et al. A Python library for probabilistic analysis of single-cell omics data. *Nat. Biotechnol.* **40**, 163–166 (2022). 35132262 10.1038/s41587-021-01206-w

[56] Zhang Fern, X. & Brodley, C. E. Solving cluster ensemble problems by bipartite graph partitioning. *ICML ‘04: Proceedings of the Twenty-first International Conference on Machine Learning* (eds Greiner, R. & Schuurmans, D.) 36 (ACM, 2004).

[57] Peng, M. et al. Cell type hierarchy reconstruction via reconciliation of multi-resolution cluster tree. *Nucleic Acids Res.* **49**, e91 (2021). 34125905 10.1093/nar/gkab481 PMC8450107

[58] Liang, S., Liang, Q., Chen, R. & Chen, K. Stratified test accurately identifies differentially expressed genes under batch effects in single-cell data. *IEEE/ACM Trans. Comput. Biol. Bioinform.* **18**, 2072–2079 (2021). 34232885 10.1109/TCBB.2021.3094650 PMC8717684

[59] Yates, A. D. et al. Ensembl 2020. *Nucleic Acids Res.* **48**, D682–D688 (2020). 31691826 10.1093/nar/gkz966 PMC7145704

[60] UniProt, C. UniProt: the universal protein knowledgebase in 2021. *Nucleic Acids Res.* **49**, D480–D489 (2021). 33237286 10.1093/nar/gkaa1100 PMC7778908

[61] Mai, J. K., Majtanik, M. & Paxinos, G. *Atlas of the Human Brain* 4th edn (Academic, 2015).

[62] Choudhary, S. & Satija, R. Comparison and evaluation of statistical error models for scRNA-seq. *Genome Biol.* **23**, 27 (2022). 35042561 10.1186/s13059-021-02584-9 PMC8764781

[63] Gardner, E. J. et al. Damaging missense variants in IGF1R implicate a role for IGF-1 resistance in the etiology of type 2 diabetes. *Cell Genom.* **2**, None (2022). 36530175 10.1016/j.xgen.2022.100208 PMC9750938

[64] McLaren, W. et al. The Ensembl variant effect predictor. *Genome Biol.* **17**, 122 (2016). 27268795 10.1186/s13059-016-0974-4 PMC4893825

[65] Karczewski, K. J. et al. The mutational constraint spectrum quantified from variation in 141,456 humans. *Nature* **581**, 434–443 (2020). 32461654 10.1038/s41586-020-2308-7 PMC7334197

[66] Rentzsch, P., Witten, D., Cooper, G. M., Shendure, J. & Kircher, M. CADD: predicting the deleteriousness of variants throughout the human genome. *Nucleic Acids Res.* **47**, D886–D894 (2019). 30371827 10.1093/nar/gky1016 PMC6323892

[67] Loh, P. R. et al. Efficient Bayesian mixed-model analysis increases association power in large cohorts. *Nat. Genet.* **47**, 284–290 (2015). 25642633 10.1038/ng.3190 PMC4342297

[68] Yang, J., Lee, S. H., Goddard, M. E. & Visscher, P. M. GCTA: a tool for genome-wide complex trait analysis. *Am. J. Hum. Genet.* **88**, 76–82 (2011). 21167468 10.1016/j.ajhg.2010.11.011 PMC3014363

