# Author Correction: A comprehensive spatio-cellular map of the human hypothalamus

**DOI:** 10.1038/s41586-025-08826-1

**Published:** 2025-02-25

**Authors:** John A. Tadross, Lukas Steuernagel, Georgina K. C. Dowsett, Katherine A. Kentistou, Sofia Lundh, Marta Porniece, Paul Klemm, Kara Rainbow, Henning Hvid, Katarzyna Kania, Joseph Polex-Wolf, Lotte Bjerre Knudsen, Charles Pyke, John R. B. Perry, Brian Y. H. Lam, Jens C. Brüning, Giles S. H. Yeo

**Affiliations:** 1 Medical Research Council Metabolic Diseases Unit, Institute of Metabolic Science-Metabolic Research Laboratories, University of Cambridge, Cambridge, UK; 2 Cambridge Genomics Laboratory, Cambridge University Hospitals NHS Foundation Trust, Cambridge, UK; 3 Department of Histopathology, Cambridge University Hospitals NHS Foundation Trust, Cambridge, UK; 4 Department of Neuronal Control of Metabolism, Max Planck Institute for Metabolism Research, Cologne, Germany; 5 Medical Research Council Epidemiology Unit, Institute of Metabolic Science, University of Cambridge, Cambridge, UK; 6 Research & Early Development, Novo Nordisk A/S, Måløv, Denmark; 7 Genomics Core, Cancer Research UK Cambridge Institute, Cambridge, UK; 8 Cologne Excellence Cluster on Cellular Stress Responses in Aging-Associated Diseases (CECAD) and Center for Molecular Medicine Cologne (CMMC), University of Cologne, Cologne, Germany; 9 Center for Endocrinology, Diabetes and Preventive Medicine (CEDP), University Hospital Cologne, Cologne, Germany; 10 National Center for Diabetes Research (DZD), Neuherberg, Germany

**Keywords:** Neural circuits, Obesity

Correction to: *Nature* 10.1038/s41586-024-08504-8 Published online 5 February 2025

In the version of the article initially published, the EGA accession number was incorrect in the Data availability section and has now been amended to EGAD50000000997 in the HTML and PDF versions of the article.

