# Supplementary material for: A comprehensive spatio-cellular map of the human hypothalamus
Source: Nature. 2025 Feb 5;639(8055):708–16. doi: 10.1038/s41586-024-08504-8 (PMC11922758; doi:10.1038/s41586-024-08504-8)
Supplement: Supplementary file 1 — A guide to Supplementary Tables 1–20 (tables supplied separately). [file 41586_2024_8504_MOESM1_ESM.pdf]

nature portfolio

https://doi.org/10.1038/s41586-024-08504-8

Supplementary information

A comprehensive spatio-cellular map of the human hypothalamus

In the format provided by the authors and unedited

Supplementary Tables Guide (tables supplied separately)

Supplementary Table 0: Dictionary
Table and column legend for each of the subsequent tables.

Supplementary Table 1: Donor overview
Donor information for Tadross et al. snRNAseq and spatial transcriptomics data and Siletti et al. snRNAseq data.

Supplementary Table 2: Sample overview
Sample information and quality control statistics for snRNAseq data

Supplementary Table 3: Cell types for scvi evaluation metric
Cell type signatures used to generate validation types for scvi tuning prior to final clustering

Supplementary Table 4: Scvi model tuning results
Output of scIntegration pipeline containing sample mixing and cell type purity scores for different parameter combinations

Supplementary Table 5: Edgelist of hierachcical clustering tree
Describing the relationship of all nodes (clusters on different levels) in the cluster tree

Supplementary Table 6: Annotations of hierachcical clustering tree
Id and fully annoated name for clusters from all levels. Also includes top marker genes per cluster, as well as covariate(Dataset,Donor,Sample) distribution in each cluster.

Supplementary Table 7: Cluster marker genes (global)
Up to top 100 significant marker genes of a cluster based on differential gene expression vs all other clusters (within the same C0 class). For example neuronal clusters are compared against other neuronal clusters. C0 markers are based on comparison against other C0 classes.

Supplementary Table 8: Cluster marker genes (Sibling)
Up to top 100 significant marker genes of a cluster based on differential gene expression only vs other clusters with the same parent cluster (siblings).

Supplementary Table 9: Comparison with clustering results from Herb et al.
Comparison of levels C2-H53, C3-H108, C4-H369 including the best corresponding cluster(s) from this study and the study by Herb et al.

Supplementary Table 10: Spatial atlas location of slices
Identification of atlas location of each spatial transcriptomics section using the Paxinos Atlas of the Human Brain 4th Edition.

Supplementary Table 11: Mean region abundance C2/C3
Mean abundance value (based on cell2location) of each C3 neuronal and C2 non-neuronal snRNAseq cluster in each spatial transcriptomics regional cluster.

Supplementary Table 12: Region abundance assignment
For all C2-C4 snRNAseq cluster clusters their assigned spatial region, based on the best matching spatial transcriptomics regional cluster(s) and associated statistics.

Supplementary Table 13: Mean region abundance C4
Mean abundance value (based on cell2location) of each C4 snRNAseq cluster in each spatial transcriptomics regional cluster.

Supplementary Table 14: Human to mouse comparison
Cross-species comparison table after final filtering with human C4 neuronal clusters and their matching mouse C465 (mouse HypoMap) neuronal clusters and their pearson correlation.

Supplementary Table 15: Melanocortin cell types
Cell type overview of human and mouse neuronal clusters expressing MC3R or MC4R including the average expression of receptors and selected marker genes.

Supplementary Table 16: Incretin cell types
Cell type overview of human and mouse neuronal clusters expressing GLP1R or GIPR including the average expression of receptors and selected marker genes.

Supplementary Table 17: Magma results
C4 cluster enrichment in the BMI GWAS signals.

Supplementary Table 18: Effector Genes
Effector genes based on the MAGMA gene associations derived from the BMI GWAS signals.

Supplementary Table 19: Exome Signals
Results of rare-variant burden tests towards BMI in the UK Biobank study.

Supplementary Table 20: Regional abbreviations
List of Regional abbreviations used in the study.
