# Supplementary material for: A comprehensive spatio-cellular map of the human hypothalamus
Source: Nature. 2025 Feb 5;639(8055):708–16. doi: 10.1038/s41586-024-08504-8 (PMC11922758; doi:10.1038/s41586-024-08504-8)
Supplement: Supplementary file 2 — Reporting Summary [file 41586_2024_8504_MOESM2_ESM.pdf]

nature portfolio

Corresponding author(s): Giles Yeo and Jens Bruning

Last updated by author(s): Jan 29, 2025

Reporting Summary

Nature Portfolio wishes to improve the reproducibility of the work that we publish. This form provides structure for consistency and transparency in reporting. For further information on Nature Portfolio policies, see our Editorial Policies and the Editorial Policy Checklist.

Statistics

For all statistical analyses, confirm that the following items are present in the figure legend, table legend, main text, or Methods section.

n/a	Confirmed	
☐	☒	The exact sample size (*n*) for each experimental group/condition, given as a discrete number and unit of measurement
☒	☐	A statement on whether measurements were taken from distinct samples or whether the same sample was measured repeatedly
☐	☒	The statistical test(s) used AND whether they are one- or two-sided<br>*Only common tests should be described solely by name; describe more complex techniques in the Methods section.*
☐	☒	A description of all covariates tested
☐	☒	A description of any assumptions or corrections, such as tests of normality and adjustment for multiple comparisons
☒	☐	A full description of the statistical parameters including central tendency (e.g. means) or other basic estimates (e.g. regression coefficient) AND variation (e.g. standard deviation) or associated estimates of uncertainty (e.g. confidence intervals)
☐	☒	For null hypothesis testing, the test statistic (e.g. *F*, *t*, *r*) with confidence intervals, effect sizes, degrees of freedom and *P* value noted<br>*Give P values as exact values whenever suitable.*
☒	☐	For Bayesian analysis, information on the choice of priors and Markov chain Monte Carlo settings
☒	☐	For hierarchical and complex designs, identification of the appropriate level for tests and full reporting of outcomes
☐	☒	Estimates of effect sizes (e.g. Cohen's *d*, Pearson's *r*), indicating how they were calculated

Our web collection on statistics for biologists contains articles on many of the points above.

Software and code

Policy information about availability of computer code

Data collection

No specific software was used for data collection.

Genetic study based on the UK Biobank was performed using application 9905.

Data analysis

All genomics data, unless otherwise specified are based on Human genome build GRCh38

The list of software below:
Python 3.10.8 - 3.10.12
scvi 0.19.0
scanpy 1.9.8
pandas 1.4.4
numpy 1.26.4
cell2location 0.1.2
cellbender 0.1 - 0.2
cellex 1.2.2
CELLECT 1.3.0

R 4.3.1
future.apply 1.11.1-9001
future 1.33.1-9009
pbapply 1.7-2

Matrix 1.6-1.1
scUtils 0.0.1
magrittr 2.0.3
igraph 1.5.1
treeio 1.26.0
ggh4x 0.2.6
scales 1.2.1
edgeR 4.0.16
limma 3.58.1
ggtree 3.10.1
lubridate 1.9.3
forcats 1.0.0
stringr 1.5.0
dplyr 1.1.3
purrr 1.0.2
readr 2.1.4
tidyr 1.3.0
tibble 3.2.1
ggplot2 3.4.4
tidyverse 2.0.0
SeuratObject 4.1.4
Seurat 4.4.0
RcppAnnoy 0.0.22

cellranger version 4-5
spaceranger 2
bolt-lmm 2.3.6

Git repositories:
https://github.com/lsteuernagel/HYPOMAP
https://github.com/lsteuernagel/scIntegration
https://github.com/georgiedowsett/HYPOMAP
https://github.com/mrcepid-rap

Please refer to the Methods for details

For manuscripts utilizing custom algorithms or software that are central to the research but not yet described in published literature, software must be made available to editors and reviewers. We strongly encourage code deposition in a community repository (e.g. GitHub). See the Nature Portfolio guidelines for submitting code & software for further information.

Data

Policy information about availability of data

All manuscripts must include a data availability statement. This statement should provide the following information, where applicable:

- Accession codes, unique identifiers, or web links for publicly available datasets
- A description of any restrictions on data availability
- For clinical datasets or third party data, please ensure that the statement adheres to our policy

The HYPOMAP snRNA-seq data is available in an interactive cellxgene viewer at https://cellxgene.cziscience.com/collections/d0941303-7ce3-4422-9249-cf31eb98c480. Additionally, the Seurat and anndata objects of HYPOMAP (snRNA-seq and spatial transcriptomics) and the scvi model, which are required to reproduce figures and to project new data, are deposited at University of Cambridge's Apollo Repository (https://doi.org/10.17863/CAM.111988). The newly generated human snRNA-seq are deposited at the European Genome-Phenome Archive (https://ega-archive.org/) under accession numbers EGAD50000000997 . The spatial transcriptomics data are available from Gene Expression Omnibus (GEO), accession number GSE278848.

All data used in genetic association analyses are available from the UK Biobank upon application (https://www.ukbiobank.ac.uk).

Research involving human participants, their data, or biological material

Policy information about studies with human participants or human data. See also policy information about sex, gender (identity/presentation), and sexual orientation and race, ethnicity and racism.

Reporting on sex and gender	No sex specific analyses are included. Individual level sex data is included in the analyses..
Reporting on race, ethnicity, or other socially relevant groupings	Details for the UKBiobank have been described elsewhere and could be found via the following link:<br>UKBB: https://www.ukbiobank.ac.uk/
Population characteristics	Details are reported in the supplementary data. Age, sex, BMI, brain weight, post-mortem interval and cause of death are available covariate for the Post-mortem brains.<br>UKBB: https://www.ukbiobank.ac.uk/
Recruitment	Post-mortem brains: Subjects were approached in life for written consent for brain banking as per each bank's local protocols.

	Genetic Studies using the UK Biobank: People aged 40-69 years who were registered with the National Health Service and living up to 25 miles from one of the 22 study assessment centres were invited to participate in 2006-2010. Overall about 9.2 million invitations were mailed to recruit 503,325 participants (a response rate of 5.47%).
Ethics oversight	Post-mortem brains: Anonymised human samples were obtained from the MRC Brain Bank Network, in line with each bank's (Cambridge Brain Bank, MRC London Neurodegenerative Diseases Brain Bank, South West Dementia Brain Bank and Edinburgh Brain and Tissue Bank) and University of Leipzig Medical Center - Institute of Anatomy, Research Ethics Committee approval. Subjects were approached in life for written consent for brain banking, and all tissue donations were collected and stored following legal and ethical guidelines.<br><br>UKBioBank: National Research Ethics Service Committee North West-Haydock and all study proceures were performed in accordance with the World Medical Association Declaration of Helsinki ethnical principles for medical research.

Note that full information on the approval of the study protocol must also be provided in the manuscript.

Field-specific reporting

Please select the one below that is the best fit for your research. If you are not sure, read the appropriate sections before making your selection.

☒ Life sciences ☐ Behavioural & social sciences ☐ Ecological, evolutionary & environmental sciences

For a reference copy of the document with all sections, see nature.com/documents/nr-reporting-summary-flat.pdf

Life sciences study design

All studies must disclose on these points even when the disclosure is negative.

Sample size	Post-mortem brains: All samples matching inclusion criteria, listed in the MRC brain bank network database, and for which tissue was acquirable were included in the snRNAseq study. smFISH sample size was determined by the availability of suitably fixed tissue from the relevant brain bank.<br><br>For UK Biobank analysis: we used every available participant who had both phenotypic data for the relevant measure and genotype data that was available at the time of submission. Individual level data was used from the UKBB (N=488,221),
Data exclusions	QC exclusion criteria for snRNAseq data are detailed in the manuscript, nuclei with less than 800 UMI, > 10% MT percent RNA which usually represent bad quality cells, and doublets were removed from the analysis. These are typical criterion used for single cell data analysis.<br>UKBB genetic analysis: Individuals who did not have genotype data were excluded from genetic association analyses. Ancestry outliers and individuals who were not of European ancestry were excluded from analyses.
Replication	The snRNAseq data presented is from two independent laboratories. Conclusions are drawn where both datasets are concordant. The smFISH data replicates previous data published by our lab on the distribution of GIPR and GLP1R in the human hypothalamus. ST is collected from 9 sections and 7 donors, all replications were successful and presented in the manuscripts.
Randomization	All genetic associations were controlled for age, sex (except for sex-specific phenotypes), population stratification, place and time of sample collection where appropriate.
Blinding	Post-mortem brains: Brains were selected based on the clinical criteria as described in the methods. This is also a descriptive study where we did not perform any direct comparisons between different brain samples.<br><br>For genetic associations analyses blinding does not apply as information on genotype and traits described were needed to perform analysis.

Reporting for specific materials, systems and methods

We require information from authors about some types of materials, experimental systems and methods used in many studies. Here, indicate whether each material, system or method listed is relevant to your study. If you are not sure if a list item applies to your research, read the appropriate section before selecting a response.

Materials & experimental systems

n/a	Involved in the study
☒	☐ Antibodies
☒	☐ Eukaryotic cell lines
☒	☐ Palaeontology and archaeology
☒	☐ Animals and other organisms
☒	☐ Clinical data
☒	☐ Dual use research of concern
☒	☐ Plants

Methods

n/a	Involved in the study
☒	☐ ChIP-seq
☒	☐ Flow cytometry
☒	☐ MRI-based neuroimaging

Plants

Seed stocks

Report on the source of all seed stocks or other plant material used. If applicable, state the seed stock centre and catalogue number. If plant specimens were collected from the field, describe the collection location, date and sampling procedures.

Novel plant genotypes

Describe the methods by which all novel plant genotypes were produced. This includes those generated by transgenic approaches, gene editing, chemical/radiation-based mutagenesis and hybridization. For transgenic lines, describe the transformation method, the number of independent lines analyzed and the generation upon which experiments were performed. For gene-edited lines, describe the editor used, the endogenous sequence targeted for editing, the targeting guide RNA sequence (if applicable) and how the editor was applied.

Authentication

Describe any authentication procedures for each seed stock used or novel genotype generated. Describe any experiments used to assess the effect of a mutation and, where applicable, how potential secondary effects (e.g. second site T-DNA insertions, mosiacism, off-target gene editing) were examined.
